# Identification and validation of NCOA5-ZCCHC3/FGF22 axis as a prognostic marker in lung adenocarcinoma migration and invasion

**DOI:** 10.1038/s41419-026-09127-9

**Published:** 2026-07-25

**Authors:** Yiran Yu, Wen Jin, Erdun Chaogetu, Xiaoli Zhang, Xi Liu, Yahan Gao, Feng Li, Rila Su

**Affiliations:** 1 Graduate School of Inner Mongolia Medical University, Hohhot, China; 2 Clinical Medical Research Center of Inner Mongolia People’s Hospital, Hohhot, China; 3 Thoracic Surgery Department of Inner Mongolia People’s Hospital, Hohhot, China; 4 Oncology Department of Inner Mongolia People’s Hospital, Hohhot, China

**Keywords:** Non-small-cell lung cancer, Translational research

## Abstract

Lung adenocarcinoma (LUAD), the most common subtype of lung cancer, is associated with substantial global mortality. Nuclear receptor coactivator 5 (NCOA5) has been implicated in several malignancies; however, its functional role and regulatory mechanisms in LUAD remain largely unknown. In this study, NCOA5 expression was evaluated in 94 paired LUAD and adjacent tissues using immunohistochemistry, RT-PCR, and Western blotting. Functional analyses were conducted using shRNA knockdown, CRISPR-mediated knockout, and overexpression models to assess the effects of NCOA5 on LUAD cell proliferation, migration, invasion, apoptosis, cell cycle progression, and organoid formation. Xenograft models were used to validate tumorigenicity in vivo. IP-MS and co-immunoprecipitation identified NCOA5-interacting proteins, while ChIP-seq and dual-luciferase assays interrogated downstream transcriptional regulation. NCOA5 was significantly upregulated in LUAD tissues and associated with advanced stage, poor differentiation, and reduced overall survival, serving as an independent prognostic factor (*P* < .040). NCOA5 knockdown inhibited LUAD cell proliferation, migration, invasion, induced G0/G1 arrest, promoted apoptosis, reduced organoid formation, and suppressed xenograft growth, whereas NCOA5 overexpression produced the opposite effects. Among 39 candidate interacting proteins identified by IP-MS, ZCCHC3 was validated as a direct NCOA5-binding partner. ZCCHC3 depletion phenocopied NCOA5 loss and reversed NCOA5-induced proliferation, migration, invasion, and colony formation, supporting a functional NCOA5–ZCCHC3 interaction. ChIP-seq analysis identified fibroblast growth factor 22 (FGF22) as a direct transcriptional target of NCOA5. FGF22 was markedly downregulated in LUAD tissues and higher FGF22 expression was associated with improved patient survival. NCOA5 suppressed FGF22 transcription, while NCOA5 inhibition increased FGF22 expression. Functionally, FGF22 knockdown enhanced LUAD aggressiveness, whereas FGF22 restoration abrogated the oncogenic effects of NCOA5 in vitro and in vivo. Collectively, these findings identify a previously unrecognized NCOA5–ZCCHC3/FGF22 axis that drives LUAD progression and provide new mechanistic insight into the role of NCOA5 in LUAD biology.

## Introduction

Lung cancer remains the leading cause of cancer-related mortality [1, 2] worldwide, responsible for an estimated 1.8 million deaths annually. Lung adenocarcinoma (LUAD), the most common histological subtype, accounts for nearly 40% of all lung cancer cases [3]. Despite progress in early detection and therapeutic strategies, the prognosis for LUAD remains unsatisfactory, largely due to late-stage diagnosis, high metastatic potential, and marked tumor heterogeneity [4, 5]. Therefore, elucidating the molecular mechanisms driving LUAD progression is critical for identifying reliable diagnostic biomarkers and developing effective targeted therapies [5–7].

Nuclear receptor coactivators are essential regulators of gene transcription that modulate the activity of nuclear hormone receptors and multiple transcription factors. Nuclear receptor coactivator 5 (NCOA5), also known as Coactivator-Independent of AF-2 Function (CIA), is an atypical coactivator that interacts with estrogen receptors and participates in both transcriptional activation and repression of target genes [8]. NCOA5 is broadly expressed across human tissues and has been implicated in diverse physiological and pathological processes [9]. Increasing evidence suggests a role for NCOA5 in tumorigenesis: dysregulated NCOA5 expression has been reported in hepatocellular carcinoma [10–18], thyroid cancer [19], esophageal cancer [20], osteosarcoma [21], and cervical cancer [22]. Elevated NCOA5 levels have additionally been associated with poor prognosis in breast [23–25], ovarian [26], and colorectal cancer [27], supporting its potential oncogenic function.

Despite accumulating evidence implicating NCOA5 in multiple malignancies, its clinical relevance, interacting partners, and downstream transcriptional mechanisms in LUAD remain poorly understood. In particular, it is unclear whether NCOA5 promotes LUAD progression through specific protein interaction networks and target gene regulation. In this study, we investigated the expression and prognostic significance of NCOA5 in LUAD, and further explored its molecular mechanism by integrating functional assays, immunoprecipitation coupled with mass spectrometry (IP-MS), co-immunoprecipitation, and ChIP-seq analysis. Our findings identify a previously unrecognized NCOA5–zinc finger CCHC-type containing 3 (ZCCHC3)/FGF22 regulatory axis that contributes to LUAD proliferation, migration, invasion, and tumor growth.

## Materials and methods

### Reagents

All antibodies used are listed in Table S1. All reagents (including chemicals, oligonucleotides, animals, deposited data, and other materials) used are described in Table S2. All primers are described in Table S3.

### Patients and follow-up

Patients with biopsy-confirmed LUAD who underwent primary surgical resection at Inner Mongolia People’s Hospital (Jan–Dec, 2008) were included. Patients who received neoadjuvant therapy were excluded. Tumor staging followed AJCC 8th-edition TNM criteria. Resected tissues were used for immunohistochemistry (IHC), RNA, and protein analyses. Patients were followed every three months in the first postoperative year, every six months for years 2–5, and annually thereafter. Overall survival (OS) was defined from surgery to death or last follow-up. Ethics approval and informed consent were obtained.

### Immunohistochemistry

A portion of the tissue was fixed in 10% neutral-buffered formalin and embedded in paraffin. Sections of the paraffin-embedded tissue, each 5 µm thick, were dewaxed and rehydrated to facilitate antigen retrieval, which was carried out in a microwave oven for 15–20 min. The specimens were then submerged in a 3% hydrogen peroxide solution for 10 min to eliminate endogenous peroxidase activity, followed by a 10 min incubation with 5% goat serum. Next, the samples were incubated overnight at 4 °C with the NCOA5 antibody. Afterward, the sections were incubated at 25 °C with a biotin-labeled secondary antibody for 10–30 min. Finally, DAB staining was applied to the tissue sections, allowing for the visualization of specific NCOA5 protein expression, which was then examined under a microscope after a coverslip was applied. Under a high-power microscope at ×400 magnification, 100 cells were counted at each site, resulting in a total of 400 cells per case. Staining intensity and percentage of positive cells were semiquantitatively evaluated by two pathologists, and expression levels were scored: 0 points for no positive cells, 1 point for 1–33% positivity, 2 points for 34–66% positivity, 3 points for ≥67% positivity and classified as low (0–3) or high [4–6].

### Western blotting and RT-PCR

Fresh-frozen tissues and LUAD cell lines were processed for protein and RNA extraction. Protein concentration was detected using the BCA method and equal amounts of protein were mixed with SDS-PAGE loading buffer. The proteins were resolved by electrophoresis on a 10% SDS-PAGE gel and then electro transferred onto a PVDF membrane and was blocked with 5% fat-free milk for 2 h, followed by an overnight incubation at 4 °C with the respective primary antibodies NCOA5, ZCCHC3, FGF22 or GAPDH. The membrane was incubated with the appropriate secondary antibody for 1 h on next day. The PVDF membrane was exposed to ECL luminescence solution and then placed in the ImageQuantTM LAS 4000 imaging system for photography.

cDNA was synthesized from the extracted RNA through reverse transcription using a PrimeScript RT kit. Subsequently, the cDNA was subjected to PCR reactions with a SYBR Premix Ex Taq kit on an ABI 7500 system, followed by agarose gel electrophoresis analysis. The PCR conditions were as follows: an initial denaturation at 95 °C for 10 min, followed by 40 cycles at 95 °C for 15 s and 64 °C for 30 s. Gene expression was quantified by RT-PCR using SYBR Green and the 2-ΔΔCt method.

### Cell culture and transfection

LUAD cell lines (A549, H1299, H1975, BEAS-2B) were obtained from the American Type Culture Collection and cultured in DMEM medium. This medium was supplemented with 5% fetal bovine serum, 100 KU/L of penicillin, and 100 mg/L of streptomycin. Cells in optimal growth conditions were digested into single-cell suspensions using 0.25% trypsin with 1 mM EDTA and placed in an incubator at 37 °C with 5% CO_2_ for growth. For transfection, cells were seeded in 35-mm plates and transfected using Lipofectamine 2000 when they reached 60–70% confluence. Monoplasmids and empty vectors were introduced into the cells and knockdown/knockout/overexpression were performed using Lipofectamine 2000 or Lipo8000.

### Cell proliferation, colony formation, and flow cytometry

Cell proliferation was assessed using CCK-8 assays. A total of 2 × 10³ cells were seeded into each well of a 96-well culture plate. At 0, 24, 48, 72, 10 μL of CCK8 solution was added to each well and incubated at 37 °C for 4 h. After incubation, the absorbance was measured using the Bio-Rad 680 microplate reader.

A total of 1000 cells was seeded in a 6-well culture plate, were cultured in a 37 °C incubator with 5%CO_2_ and saturated humidity for 2 weeks. Once visible clones appeared, the culture process was terminated. The cells were fixed using 5 mL of 4% paraformaldehyde and stained with Giemsa.

In total, 2 × 10^5^ cells were inoculated into a 35-mm culture plate until they formed a single cell layer. Cells were collected by centrifugation, fixed with 3 mL of 70% precooled ethanol, and stored at –20 °C overnight. Cells were washed twice with PBS, 20 µg/mL RNaseA to 500 μL 1×PBS, incubated at 37 °C for 30 min, and 50 µg/mL PI to 500 μL 1×PBS, stained in the dark at room temperature for 30 min, and then filtered through a 200-mesh sieve and tested for changes in the cell cycle. For cell apoptosis, 1 × 10^5^ cells were seeded in 6-well plates. Cell cycle and apoptosis were analyzed by PI staining or Annexin V/7-AAD flow cytometry.

### Migration and invasion

A total of 1 × 10^5^ cells were placed in the upper Transwell chamber in serum-free medium, while the lower chamber contained a complete medium with 20% FBS at 37 °C. After a 24-h incubation period, the cells that did not migrate or invade from the upper surface of the membrane were removed. The cells that successfully passed through the membrane were then fixed, stained with crystal violet, and counted under a microscope.

### Organoid culture

Primary LUAD biopsy specimens were collected in conical centrifuge tubes containing ice-cold Primary Tissue Storage Solution and maintained at 4 °C until tissue isolation procedures were initiated. Fresh tumor tissues were mechanically minced into small fragments of 1–3 mm using sterile surgical scissors. For enzymatic digestion, tissue fragments were resuspended in 10 mL of Tumor Tissue Digestion Solution in 15 mL conical tubes and incubated at 37 °C for a duration of 5–30 min, with incubation time adjusted according to tissue texture and digestion status. The digestion reaction was terminated by supplementation with fetal bovine serum to achieve a final concentration of 2% (v/v) in the digestion mixture. The cell suspension was subsequently filtered through a 100μm cell strainer to remove undigested tissue debris. The filtered single-cell suspension was centrifuged at 250 × *g* for 3 min at 4 °C, after which the supernatant was carefully aspirated, and the cell pellet was resuspended in Organoid Culture Extracellular Matrix (ECM). Aliquots of the cell-containing ECM (~30 μL per droplet) were seeded at the central bottom of each well in 24-well cell culture plates. Following complete solidification of ECM droplets, 500 μL of organoid complete culture medium was gently added to each well. The culture plates were finally incubated in a humidified incubator with 5%CO_2_ at 37 °C for subsequent organoid growth.

### Xenograft assays

Thirty nude mice (3-weeks old) were obtained from the National Laboratory Animal Center in Beijing, China. When A549 cells reached a density of 80%, they were counted following digestion with 0.25% Trypsin. After centrifugation, the cells were washed three times with Hank’s solution and then subcutaneously injected into left axilla of the nude mice at a concentration of 1 × 10^7^ cells in 0.1 ml. Mice were divided into six groups: shCtrl, shNCOA5, Ctrl, NCOA5, and NCOA5 + FGF22, and NCOA5+shZCCHC3 cells, respectively. The tumor volume was measured and recorded weekly and the xenograft tumors were excised and weighed after 6 weeks. All animal-related procedures were approved by the Ethics Committee of Inner Mongolia People’s Hospital.

### Immunoprecipitation-mass spectrometry, and Co-IP

Nuclear protein extracts were obtained from control and NCOA5 Flag-tagged cells using the NE-PER™ Nuclear and Cytoplasmic Extraction Reagents. The supernatants containing the nuclear proteins were incubated with anti-Flag antibodies for 2 h at 4 °C to selectively precipitate the NCOA5-Flag protein complexes. These complexes were purified by incubating with A/G agarose beads for 1 h at 4 °C. Afterward, the complexes were washed extensively, then purified protein samples were resolved using 10% SDS-PAGE, and the proteins were visualized in the gel using Coomassie blue staining. The resulting peptides were extracted, concentrated, and subjected to LC-MS/MS analysis using the EASY nLC 1200-Orbitrap Fusion Lumos+ETD system.

Cells were lysed in the presence of a complete protease inhibitor cocktail on ice for 30 min, then collected and antibodies specific to NCOA5, ZCCHC3, or a nonspecific control IgG were collected for 12 h at 4 °C to facilitate the formation of the antibody-protein complex. Subsequently, 20 µL of protein A/G agarose beads were introduced into the reaction mixture and incubated overnight at 4 °C. The resin was then pelleted and washed extensively, and the immunocomplexes were analyzed by western blotting.

### Chromatin immunoprecipitation and luciferase assays

A549 cells were incubated with anti-Flag antibody or anti-IgG antibody to selectively immunoprecipitate NCOA5-bound DNA fragments. These complexes were captured using magnetic beads, and the isolated DNA was purified. The purified DNA fragments, enriched for sequences associated with NCOA5 binding, were then subjected to sequencing on the NovaSeq 6000.

The FGF22 promoter sequence was cloned into the pGL4 Luciferase Reporter Vector, then seeded in 24-well plates that had been treated with polylysine to ensure optimal adhesion, allowing them to reach a confluence of 60–70%. After a 24 h incubation period, the cells were co-transfected with either 200 nM of the NCOA5 expression plasmid or siNCOA5, along with 400 ng of the wild-type FGF22 promoter plasmid. The transfection was facilitated by Lipo8000. Dual luciferase assays were performed 48 h after transfection and luciferase activity was measured with a microplate spectrophotometer.

### Statistical analysis

All experiments were performed with at least three independent biological replicates unless otherwise specified. Data were presented as mean ± standard deviation. Statistical analyses were conducted using GraphPad Prism 11.0. Comparisons between two groups were performed using a two-tailed Student’s *t* test, while comparisons among multiple groups were conducted using one-way ANOVA followed by appropriate post hoc tests. A *P* value < 0.05 was considered statistically significant. Where applicable, exact p-values are reported in the figures or text.

## Results

### NCOA5 is upregulated in LUAD and associated with poor prognosis

**Fig. 1:**
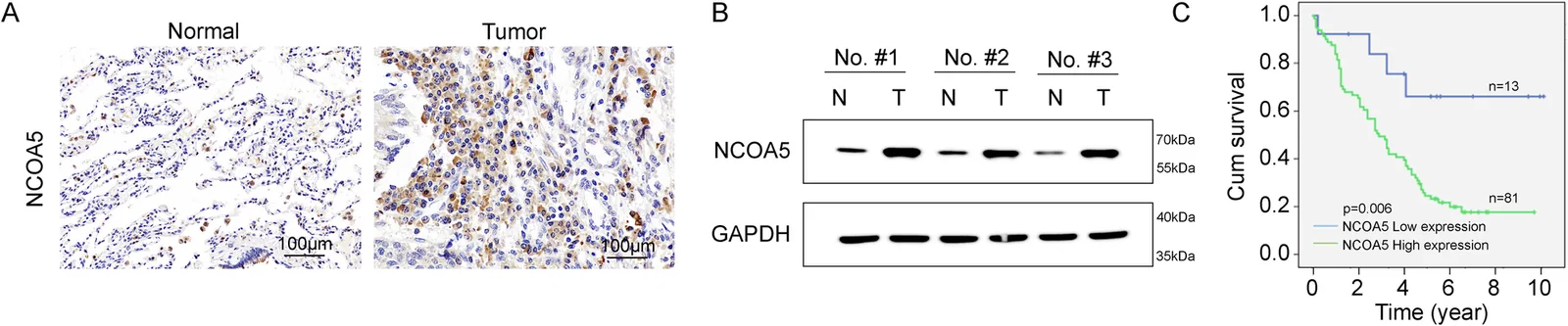
NCOA5 is upregulated in LUAD tissue and is associated with a poor prognosis. **A** IHC staining of NCOA5 in tumor tissue and normal lung tissue. T: tumor tissue and N: adjacent nontumor normal tissue; **B** the expression of NCOA5 was detected in 3 paired samples of fresh tumor tissue and normal tissues using western blotting analysis. β-actin was used as the housekeeping gene internal control; **C** correlation between overall survival and NCOA5 expression (***P = 0.006).

NCOA5 expression was examined in 94 paired LUAD and adjacent normal tissues using IHC. NCOA5 was predominantly localized to the nucleus, as indicated by strong brownish-yellow staining, and was significantly upregulated in tumor tissues (*P* < 0.001, Fig. 1A). Among them, 81(86.2%) exhibited high NCOA5 expression, while 13(13.8%) showed low expression (Table 1). Western blotting analysis further confirmed elevated NCOA5 protein levels in LUAD tissues (Fig. 1B). High NCOA5 expression was significantly associated with poor tumor differentiation (*P* = 0.010), advanced T stage (*P* = 0.025), and positive N status (*P* = 0.019) (Table 1). In contrast, NCOA5 expression showed no significant association with patient age, sex, metastatic status, tumor location, or EGFR/ALK mutation status. All patients were followed for 0 –10 years, and by the end of follow-up, 69 patients (73.4%) had died, with a mean survival time of over 4 years (52.26 months). Kaplan–Meier analysis demonstrated that patients with high NCOA5 expression had significantly shorter OS than those with low expression (*P* = 0.006, Fig. 1C). Multivariate Cox regression analysis confirmed that high NCOA5 expression was independently associated with poor prognosis in LUAD (OR = 3.46, 95% CI: 1.06–11.28) (Table 2).

**Table 1:** Correlation of clinicopathological characteristics with NCOA5 expression.

Clinical feature	NCOA5 expression		X^2^	*P* value
	(Low *N* = 13)	(High *N* = 81)		
Gender			1.516	0.218
Male	5 (38.5)	46 (56.8)		
Female	8 (61.5)	35 (43.2)		
Age			0.292	0.589
<60	6 (46.2)	31 (38.3)		
≥60	7 (53.8)	50 (61.7)		
Tumor differentiation			13.461	0.01^*^
1	3 (23.1)	1 (1.2)	VS.1	
2	6 (46.2)	56 (69.1)		
3	4 (30.8)	24 (29.6)		
T stages			9.342	0.025^*^
1	5 (38.5)	18 (22.2)	VS.1	
2	6 (46.2)	40 (49.4)		
3	1 (7.7)	23 (28.4)		
4	1 (7.7)	0 (0)		
N status			9.986	0.019^*^
0	11 (84.6)	32 (39.5)	VS.0	
1	0 (0)	22 (27.2)		
2	2 (9.6)	21 (25.9)		
3	0 (0)	6 (7.4)		
M status			1.110	0.138
0	12 (92.3)	81 (100.0)		
1	1 (7.7)	0 (0.0)		
Survival status			14.047	0.001^*^
Alive	9 (69.2)	16 (19.8)		
Death	4 (30.8)	65 (80.2)		
OS (month)	63.00 ± 37.73	39.68 ± 27.03	2.724	0.008^*^
Tumor location			0.714	0.547
Left	9 (69.2)	46 (56.8)		
Right	4 (30.8)	35 (43.2)		
EGFR mutation			0.618	0.478
0	9 (69.2)	64 (79.0)		
1	4 (30.8)	17 (21.0)		
ALK mutation			0.004	1.000
0	11 (84.6)	68 (84.0)		
1	2 (15.4)	13 (16.0)		

^*P < 0.05^

**Table 2:** Multivariate logistic regression analyses of prognostic variables.

Clinical features	B	*P* value	EXP(B)	95% CI for EXP(B) Lower Upper	
Gender (Female, 0; Male, 1)	0.215	0.406	1.240	0.747	2.057
Age (≥60, 1; <60, 0)	–0.053	0.836	0.948	0.573	1.568
Tumor location (Left lung,1; right lung,0)	0.092	0.724	1.096	0.659	1.823
TNM stage (Advanced stage III-IV)	0.752	0.074	2.123	0.931	4.844
Tumor invasive depth (T)	–0.014	0.943	0.986	0.669	1.453
Lymph node metastasis (N)	0.227	0.235	1.255	0.862	1.827
Distant metastasis (M)	1.153	0.364	3.166	0.263	38.082
NCOA5 expression (High)	1.241	0.040^*^	3.457	1.059	11.283
EGFR (FISH Positive)	0.005	0.987	1.005	0.553	1.826

*P < 0.05.

### NCOA5 promotes LUAD cell proliferation, migration, invasion, and tumor growth

**Fig. 2:**
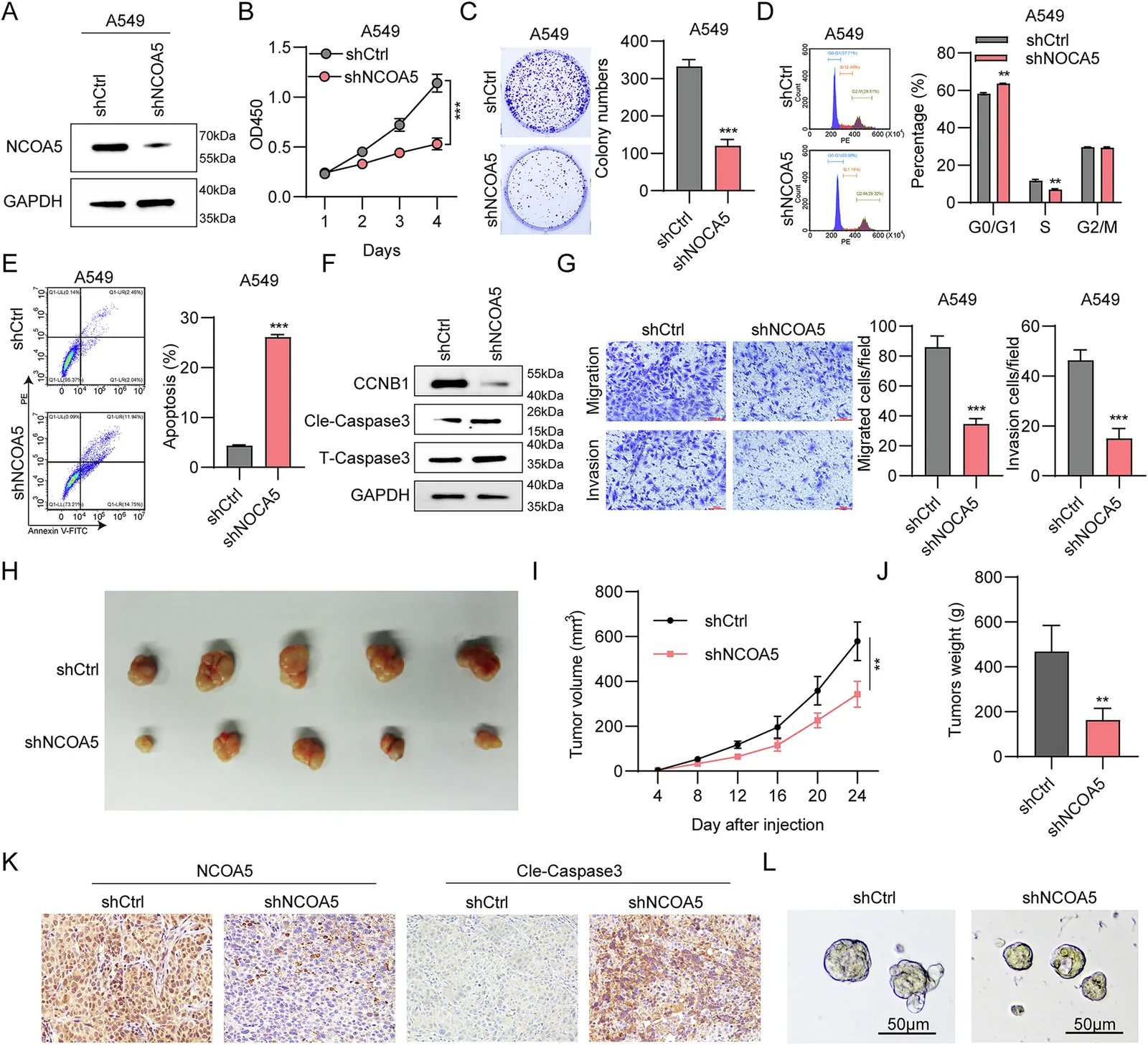
NCOA5 knockdown suppresses LUAD cell progression and tumor growth. **A** Western blotting showed NCOA5 expression in LUAD cell lines A549 transfected with shCtrl and shNCOA5; **B**, **C** CCK8 and cell colony formation assays were performed in A549 cells to compare the shNCOA5 and shCtrl groups (***P < 0.001); **D**, **E** flow cytometry assay of the cell cycle and cell apoptosis in A549 cells comparing the shNCOA5 and shCtrl groups (**P < 0.01, ***P < 0.001); **F** Western blot comparing the expression of CCNB1 and Caspase-3 in A549 cells comparing the shNCOA5 and shCtrl group; **G** migration assay and invasion assay in A549 cells comparing the shNCOA5 and shCtrl groups (***P < 0.001); **H** xenograft assay of shNCOA5 and shCtrl transfected cells injection in nude mice and the xenografted tumors; **I**, **J** the tumor volume and the tumor weight were measured, shNCOA5 compared with the vector group (**P < 0.01); **K** IHC staining of NCOA5 and Cle-Caspase-3 in shNCOA5 compared with the shCtrl group; **L** organoid models in A549 cells comparing the shNCOA5 and shCtrl groups.

**Fig. 3:**
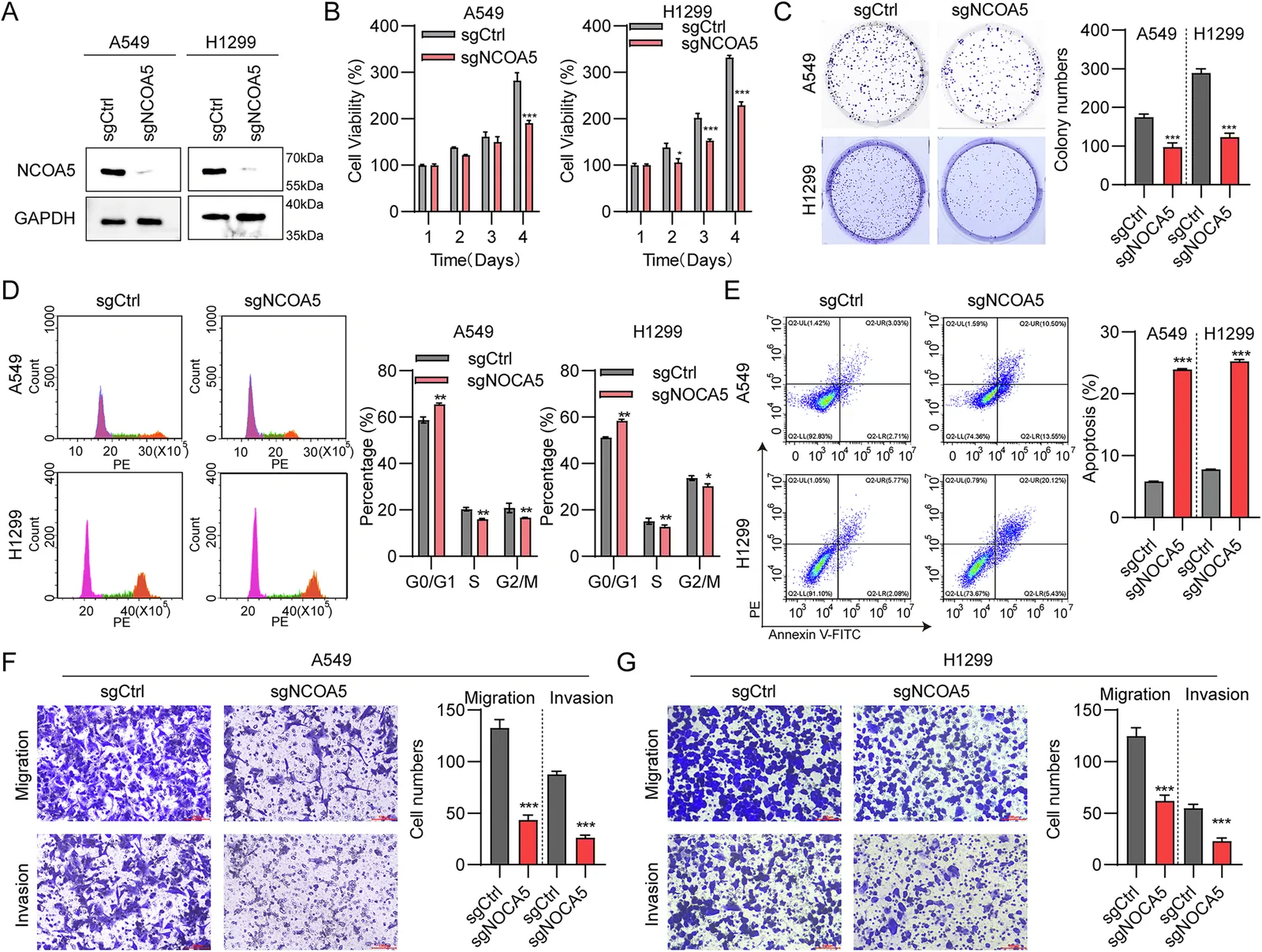
NCOA5 knockout suppresses LUAD cell progression and tumor growth. **A** Western blotting showed NCOA5 expression in LUAD cell lines A549 and H1299 transfected with sgCtrl and sgNCOA5; **B**, **C** CCK8 and cell colony formation assays were performed in A549 and H1299 cells to compare the sgNCOA5 and sgCtrl groups (*P < 0.05,**P < 0.01,***P < 0.001); **D**, **E** flow cytometry assay of the cell cycle and cell apoptosis in A549 and H1299 cells comparing the sgNCOA5 and sgCtrl groups (**P < 0.01,***P < 0.001); **F**, **G** migration assay and invasion assay in A549 and H1299 cells comparing the sgNCOA5 and sgCtrl groups (***P < 0.001).

To determine the functional role of NCOA5 in LUAD, both loss-of-function experiments were performed. NCOA5 knockdown was achieved in A549 cells using shRNA, and reduced protein expression was confirmed by Western blotting (Fig. 2A). shNCOA5 cells exhibited significantly reduced proliferation in CCK8 assays beginning on day 3 (Fig. 2B, *P* < 0.001) and formed fewer colonies than controls (Fig. 2C, *P* < 0.001). Flow cytometry analysis revealed G0/G1 arrest (Fig. 2D, 63.66% vs. 58.22%, *P* < 0.01) and a substantial increase in apoptosis (Fig. 2E, 26.2% vs. 4.5%, *P* < 0.001), accompanied by reduced CCNB1 and increased Cle-Caspase-3 expression (Fig. 2F). NCOA5 knockdown also significantly impaired cell migration and invasion (Fig. 2G, *P* < 0.001). In vivo, NCOA5-deficient A549 cells formed significantly smaller tumors in nude mice, with mean tumor volumes of 343.4 mm³ compared with 579.6 mm³ in controls at week 6 (Fig. 2H–J, *P* < 0.01). IHC staining of xenografts showed increased c-Caspase-3 expression in the shNCOA5 group (Fig. 2K), supporting enhanced apoptosis in vivo. Importantly, NCOA5 knockdown substantially reduced the formation and growth of LUAD organoids, indicating suppressed tumor-initiating potential in a three-dimensional context (Fig. 2L). Consistent phenotypes were observed in A549/H1299 cells using sgRNA-mediated NCOA5 knockout cell models, indicating that the effects of NCOA5 modulation are not restricted to a single LUAD cell line, which likewise reduced proliferation, migration and invasion and increased apoptosis (Fig. 3A–G, *P* < 0.05, *P* < 0.01, *P* < 0.001), confirming the specificity of the knockdown effects.

**Fig. 4:**
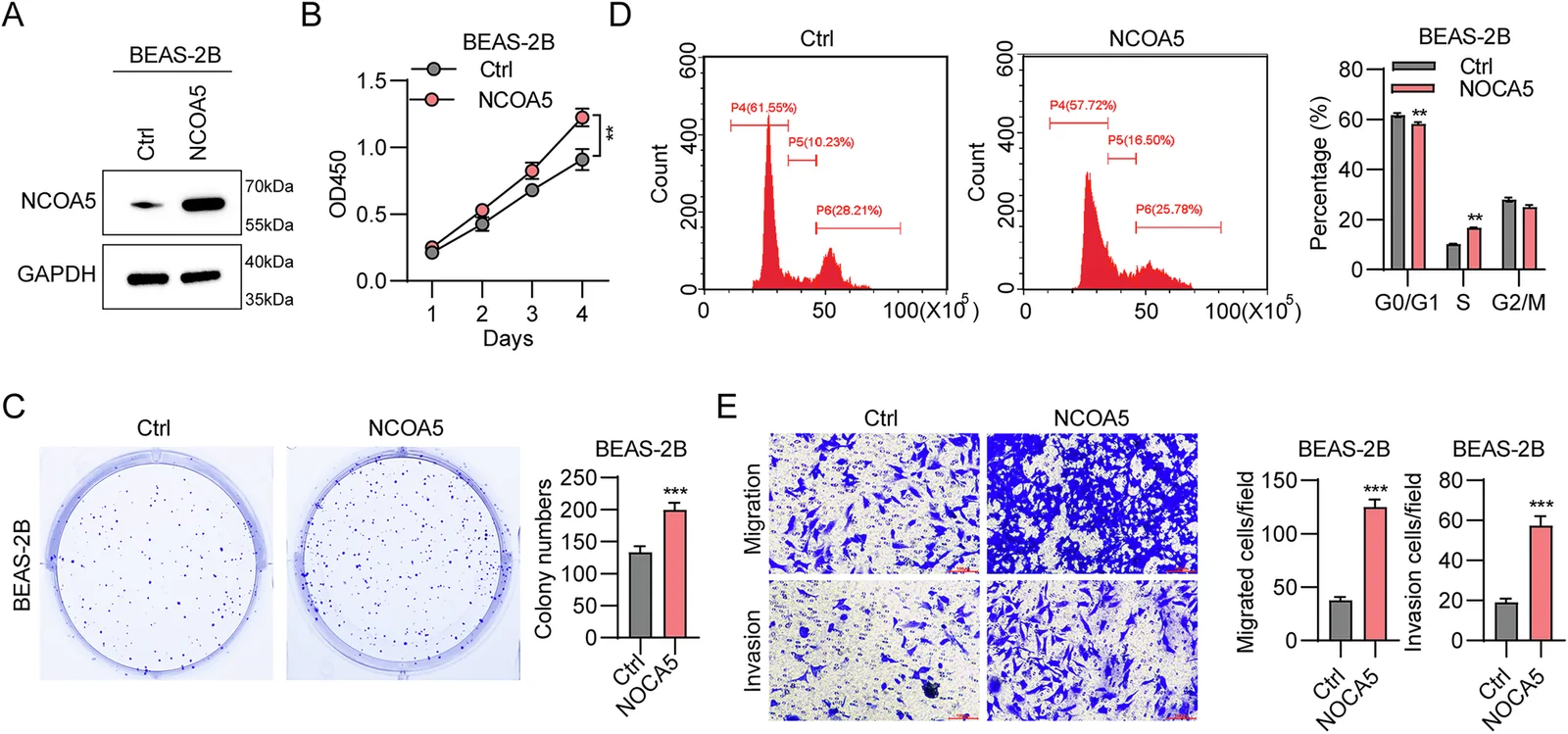
NCOA5 overexpression promotes cell proliferation, migration, and cell cycle progression of LUAD cells. **A** Western blotting showed NCOA5 expression in BEAS 2B cells transfected with Ctrl and NCOA5; **B**, **C** CCK8 and cell colony formation assays in BEAS-2B cells transfected with Ctrl andNCOA5(**P<0.01); **D** cell cycle flow cytometry assay in BEAS-2B cells transfected with Ctrl and NCOA5 (***P < 0.001); **E** migration assay and invasion assay of cell migration and invasion in BEAS-2B cells transfected with Ctrl and NCOA5 (***P < 0.001).

Complementary gain-of-function assays were performed by overexpressing NCOA5 in BEAS-2B cells, confirmed by Western blotting (Fig. 4A). NCOA5 overexpression significantly enhanced cell proliferation (Fig. 4B, *P* < 0.010) and colony formation (Fig. 4C, *P* < 0.001). Cell cycle analysis demonstrated accelerated G1–S transition (Fig. 4D, *P* < 0.010), and Transwell assays showed increased migration and invasion (Fig. 4E, *P* < 0.001). Together, these bidirectional genetic approaches provide strong evidence that NCOA5 functions as an oncogenic driver in LUAD by promoting proliferation, cell cycle progression, migration, invasion, and tumorigenicity.

### NCOA5 binds to ZCCHC3, and ZCCHC3 knockdown inhibits LUAD cell progression

**Fig. 5:**
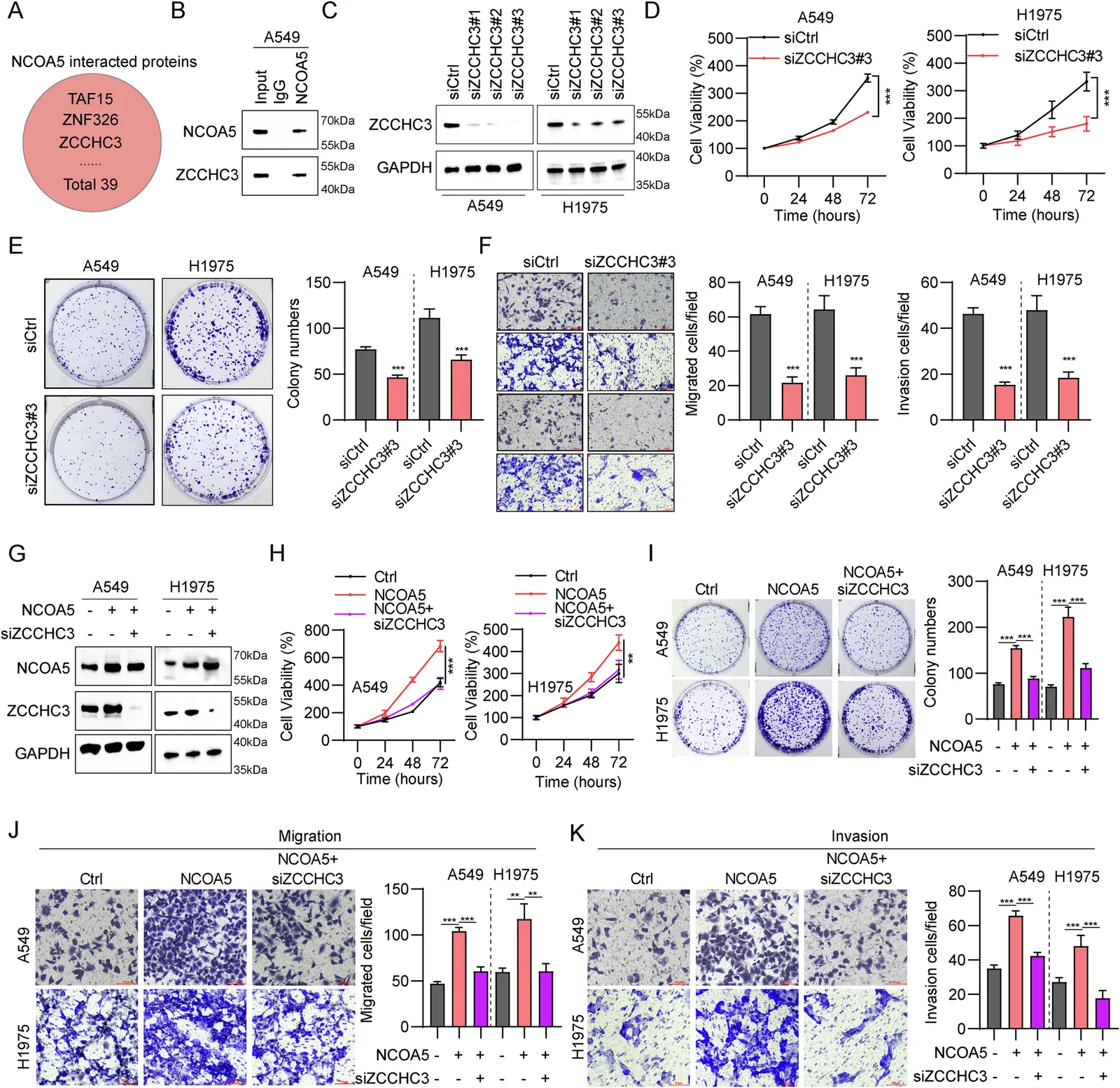
NCOA5 binds to ZCCHC3, and ZCCHC3 knockdown inhibited the progression of LUAD cells. **A** IP-MS was used to explore the proteins that interacted with NCOA5; **B** co-immunoprecipitation assay verified the interaction between ZCCHC3 and NCOA5; **C** Western blotting showed ZCCHC3 expression in A549/H1975 cells transfected with siCtrl, siZCCHC3#1, siZCCHC3#2, and siZCCHC3#3; **D**, **E** CCK-8 and cell colony formation assays in A549/H1975 cells transfected with siCtrl and siZCCHC3#3 (***P < 0.001); **F** migration assay and invasion assay of cell migration and invasion in A549/H1975 cells with siCtrl and siZCCHC3#3 (***P < 0.001); **G** Western blotting showed the expression of NCOA5 and ZCCHC3 in A549/H1975 cells transfected with Ctrl, NCOA5, and NCOA5+siZCCHC3; **H**, **I** CCK-8 and cell colony formation assays in A549/H1975 cells transfected with Ctrl, NCOA5, and NCOA5+siZCCHC3(**P < 0.01,***P < 0.001); **J**, **K** transwell assay in A549/H1975 cells transfected with Ctrl, NCOA5, and NCOA5+siZCCHC3 (**P < 0.01, ***P < 0.001).

To investigate the molecular partners through which NCOA5 exerts its oncogenic effects, IP-MS was performed. A total of 39 candidate NCOA5-interacting proteins were identified (Fig. 5A). Among these, ZCCHC3, TAF15, and ZNF326 emerged as a notable candidate. Co-immunoprecipitation assays validated this interaction, as NCOA5 was detected in anti-ZCCHC3 precipitates and ZCCHC3 was detected in anti-NCOA5 precipitates, confirming a reciprocal binding relationship (Fig. 5B). In contrast, co-IP assays using TAF15 and ZNF326 did not detect an interaction with NCOA5 (Supplementary Fig. S1). To explore the functional relevance of ZCCHC3 in LUAD, A549/H1975 cells were transfected with three independent siRNAs targeting ZCCHC3, and knockdown efficiency was verified by Western blotting (Fig. 5C). ZCCHC3 silencing significantly reduced cell proliferation, as demonstrated by CCK-8 and colony formation assays (Fig. 5D, E, *P* < 0.001). Consistently, Transwell assays revealed that ZCCHC3 knockdown markedly inhibited cell migration and invasion (Fig. 5F, *P* < 0.001). Rescue experiments further supported a functional interaction between NCOA5 and ZCCHC3. Knockdown of ZCCHC3 in NCOA5-overexpressing A549/H1975 cells (Fig. 5G), attenuated the pro-proliferative and pro-metastatic effects induced by NCOA5 overexpression, as shown by reduced cell viability (Fig. 5H, *P* < 0.001 and *P* < 0.01), decreased colony formation (Fig. 5I, *P* < 0.001), and impaired migration and invasion (Fig. 5J, K, *P* < 0.01, *P* < 0.001). Together, these findings indicate that ZCCHC3 is a critical NCOA5-binding partner and is required for the full oncogenic activity of NCOA5 in LUAD.

### FGF22 expression is regulated by NCOA5 and functions as a tumor suppressor in LUAD

**Fig. 6:**
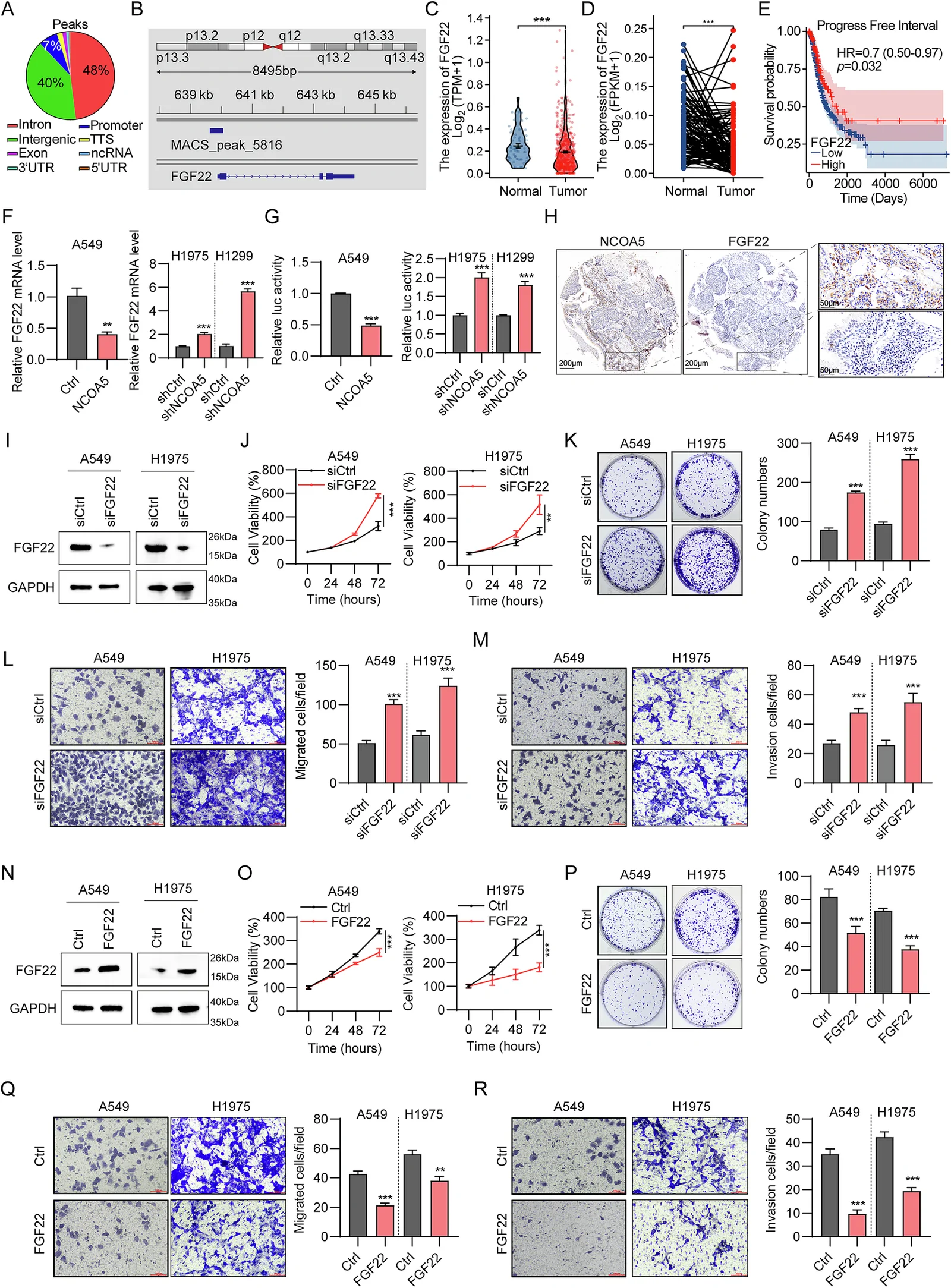
FGF22 expression was regulated by NCOA5, and FGF22 suppressed LUAD progression. **A**, **B** 7% of NCOA5 binding targets in the promoter region, and FGF22 was a direct transcriptional target of NCOA5 in this region analyzed by ChIP-Seq; **C** comparison of FGF22 low expression in LUAD samples with that in normal tissues (***P < 0.001); **D**, **E** Reduction of FGF22 expression in 94 clinical LUAD tissues, and the high expression of FGF22 is associated with a high progression-free interval (PFI) of LUAD (***P < 0.001, *P* = 0.032); **F** the impact of NCOA5 on FGF22 expression in A549 cells and H1975, H1299 cells by RT-qPCR assay (**P < 0.01, ***P < 0.001); **G** the impact of NCOA5 on FGF22 expression in A549 cells and H1975, H1299 cells luciferase reporter gene assay (***P < 0.001); **H** FGF22 downregulated in LUAD tissue, while NCOA5 was upregulated; **I** Western blotting showed FGF22 expression in A549/H1975 cells transfected with siCtrl, siFGF22; **J**, **K** CCK-8 and cell colony formation assay of cell proliferation in A549 /H1975cells transfected with siCtrl and siFGF22 (**P < 0.01,***P < 0.001); **L**, **M** transwell assay of cell migration and invasion in A549/H1975 cells transfected with siCtrl and siFGF22 (***P < 0.001); **N** Western blotting showed FGF22 overexpression in A549/H1975 cells transfected with Ctrl, FGF22; **O**, **P** CCK-8 and cell colony formation assay of cell proliferation in A549 /H1975 cells transfected with Ctrl and FGF22 (***P < 0.001); **Q**, **R** transwell assay of cell migration and invasion in A549/H1975 cells transfected with Ctrl and FGF22 (***P < 0.001).

To identify downstream transcriptional targets of NCOA5, we performed genome-wide ChIP-seq analysis. Approximately 7% of NCOA5-binding sites were located within promoter regions (Fig. 6A) and FGF22 was identified as a direct NCOA5-bound gene within this region (Fig. 6B). Analysis of LUAD samples from the TCGA dataset showed that FGF22 expression was significantly lower in tumor tissues than in normal lung tissues (Fig. 6C, *P* < 0.001). Consistently, in our cohort of 94 LUAD patients, FGF22 expression was reduced in tumor specimens compared with matched adjacent noncancerous tissues (Fig. 6D, *P* < 0.001). Higher FGF22 expression was also associated with improved progression-free interval in LUAD patients (Fig. 6E, *P* = 0.032). Importantly, analysis of paired clinical samples confirmed an inverse expression pattern between NCOA5 and FGF22, with NCOA5 upregulated and FGF22 downregulated in LUAD tumors (Fig. 6H).

To determine whether NCOA5 directly regulates FGF22 expression, we examined FGF22 levels following NCOA5 modulation. qRT-PCR analysis showed that NCOA5 overexpression suppressed FGF22 transcript levels in A549 cells, whereas NCOA5 knockout in H1975 and H1299 cells resulted in significant upregulation of FGF22 (Fig. 6F, *P* < .01). Consistently, dual-luciferase assays demonstrated reduced FGF22 promoter activity upon NCOA5 overexpression and increased promoter activity following NCOA5 knockdown (Fig. 6G, *P* < 0.001), confirming FGF22 as a transcriptional target repressed by NCOA5.

To clarify the functional consequences of FGF22 loss, we performed gain- and loss-of-function studies. FGF22 knockdown in A549/H1975 cells—validated by Western blotting (Fig. 6I) significantly promoted cell proliferation (Fig. 6J, K, *P* < 0.01, *P* < 0.001) and enhanced cell migration and invasion (Fig. 6L, M, *P* < 0.001). In contrast, overexpression of FGF22 suppressed cell proliferation (Fig. 6N, O, *P* < 0.001), reduced colony formation (Fig. 6P, *P* < 0.001), and inhibited migration and invasion in A549/H1975 cells (Fig. 6Q, R, *P* < 0.01, *P* < 0.001). Collectively, these results indicate that FGF22 is a direct transcriptional target repressed by NCOA5 and functions as a tumor suppressor in LUAD, and that NCOA5-driven FGF22 downregulation may be a key mechanism promoting LUAD progression.

### FGF22 antagonizes NCOA5-driven LUAD progression in vitro and in vivo

**Fig. 7:**
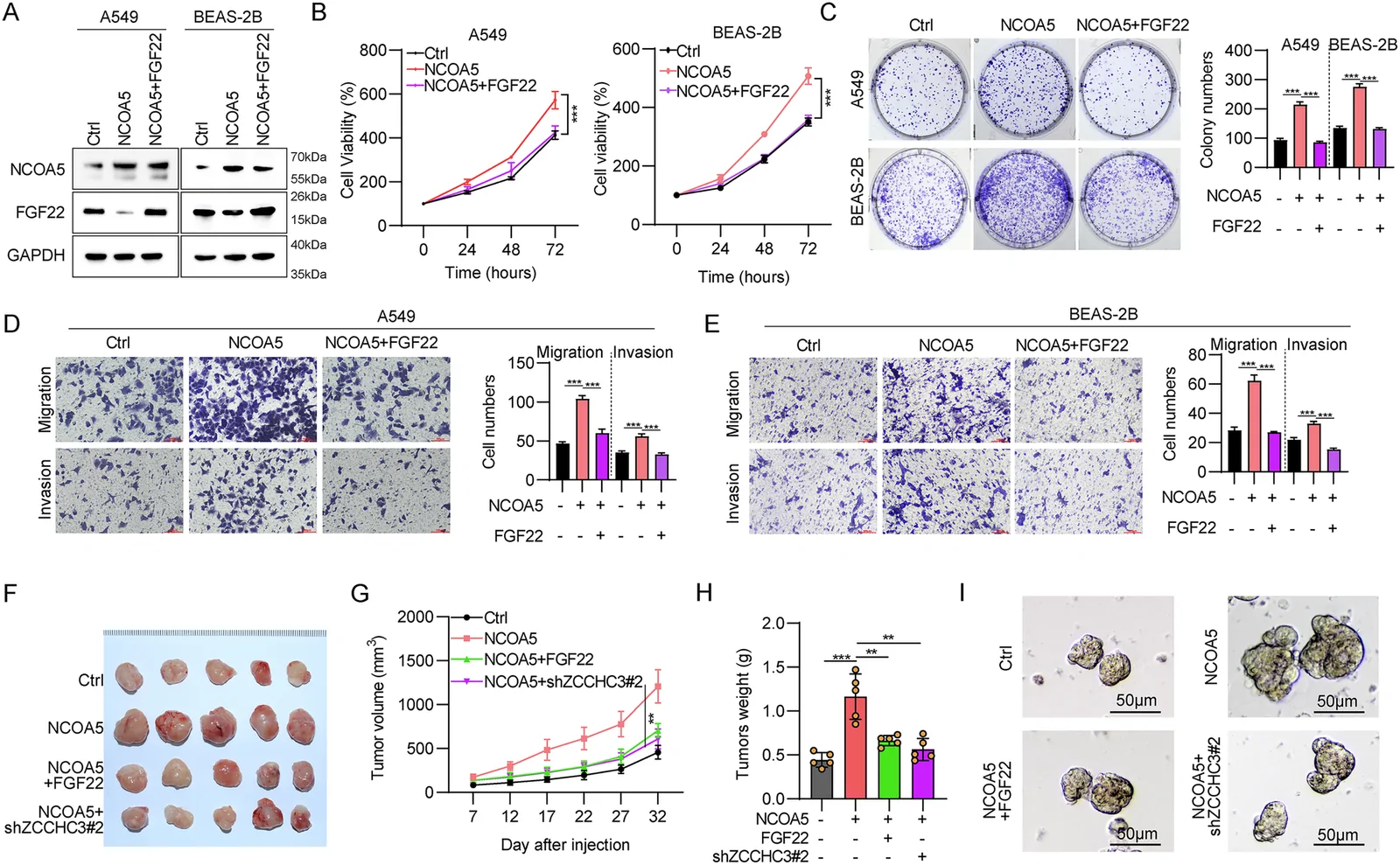
FGF22 antagonized the ability of NCOA5 to promote LUAD progression in vitro and in vivo. **A** NCOA5 overexpression (NCOA5) and NCOA5 overexpression with restored the expression of FGF22 (NCOA5 + FGF22) in A549/BEAS-2B cells; **B**, **C** FGF22 restoration abrogated the proliferative effects induced by NCOA5 overexpression in CCK-8 and colony formation assays (***P < 0.001); **D**, **E** FGF22 restoration reduces the stimulating effect of NCOA5 overexpression on A549/BEAS-2B cell migration and invasion (***P < 0.001); **F** tumorigenesis in mice injected with NCOA5 overexpression (NCOA5), NCOA5 overexpression with restoration of FGF22 (NCOA5 + FGF22), and NCOA5 with ZCCHC3 knockdown (NCOA5+shZCCHC3#2) in A549 cells; **G**, **H** the tumor volume and the tumor weight were measured in four groups, Ctrl, NCOA5, NCOA5 + FGF22 and NCOA5+shZCCHC3 group (**P < 0.01,***P < 0.001); I organoid models in A549 cells comparing the Ctrl NCOA5, NCOA5 + FGF22 and NCOA5+shZCCHC3 groups.

To determine whether FGF22 functionally counteracts NCOA5-mediated oncogenic activity, we restored FGF22 expression in A549/BEAS-2B cells overexpressing NCOA5 (NCOA5). Western blotting confirmed successful FGF22 reintroduction (Fig. 7A). Restoration of FGF22 markedly attenuated the proliferative advantage conferred by NCOA5 overexpression, as demonstrated by CCK-8 and colony formation assays (Fig. 7B-C, *P* < .001). Likewise, Transwell assays revealed that FGF22 overexpression substantially reversed the NCOA5-induced increase in cell migration and invasion (Fig. 7D, E, *P* < 0.001), indicating that FGF22 acts as a functional antagonist of NCOA5 in vitro. To determine whether ZCCHC3 participates in NCOA5-mediated repression of FGF22, we examined FGF22 expression following ZCCHC3 silencing and ZCCHC3 silencing+NCOA5 overexpression in LUAD cells. Knockdown of ZCCHC3 significantly increased FGF22 mRNA and protein levels, as demonstrated by RT-qPCR and Western blotting analyses. Consistent with our previous findings, NCOA5 overexpression markedly reduced FGF22 expression. Notably, this suppressive effect was partially reversed upon ZCCHC3 knockdown, indicating that ZCCHC3 contributes to the repression of FGF22 downstream of NCOA5 (Supplementary Fig. S2A-B). These findings suggest that the NCOA5–ZCCHC3 interaction functionally participates in the regulation of FGF22 expression, thereby supporting a mechanistic link between protein interaction and downstream transcriptional control.

To validate these findings in vivo, we established subcutaneous xenograft models using A549 cells with different genetic manipulations (Ctrl, NCOA5, NCOA5 + FGF22, and NCOA5+shZCCHC3#2). Consistent with our in vitro observations, NCOA5 overexpression significantly accelerated tumor growth compared with controls (Fig. 7F–H, *P* < 0.01). Notably, restoration of FGF22 or knockdown of ZCCHC3 both markedly suppressed NCOA5-driven tumorigenesis, indicating that inhibition of the NCOA5–ZCCHC3/FGF22 axis attenuates LUAD progression in vivo. Additionally, lung cancer organoid assays showed enhanced organoid formation following NCOA5 overexpression, whereas organoid growth was substantially reduced when ZCCHC3 was silenced or adding FGF22 in NCOA5-overexpressing cells (Fig. 7I). Collectively, these findings demonstrate that FGF22 acts as a critical downstream suppressor that counterbalances NCOA5- and ZCCHC3-mediated oncogenic activity in LUAD, both in vitro and in vivo.

## Discussion

Understanding the molecular mechanisms that drive lung cancer is essential for improving early diagnosis, risk stratification, and the development of effective targeted and immune-based therapies [28, 29]. Mechanistic studies do more than identify biomarkers: they reveal causal nodes in disease pathways that can be directly targeted or used to rationally combine therapies, and they explain heterogeneity in treatment response [28, 30, 31].

Recent literature indicates that NCOA5 exerts context-dependent roles across multiple malignancies, functioning as either an oncogene or tumor suppressor depending on tumor type and cellular context. Although dysregulated NCOA5 expression has been reported in hepatocellular carcinoma, thyroid cancer, esophageal cancer, colorectal cancer, and breast cancer, its clinical significance and mechanistic role in LUAD have remained insufficiently characterized [10, 13–27, 32]. In particular, prior studies have not established whether NCOA5 contributes to LUAD progression through a defined interacting partner and downstream transcriptional effector network. In this study, we demonstrate not only that NCOA5 is highly expressed in LUAD and associated with poor prognosis, but also that it exerts oncogenic effects through a previously uncharacterized NCOA5-ZCCHC3/FGF22 regulatory axis. A particularly novel aspect of our findings is the identification of ZCCHC3 as a functional binding partner of NCOA5 in LUAD. Although ZCCHC3 has been implicated in RNA sensing, innate immune signaling, and tumor-associated processes in other contexts, its interaction with NCOA5 and its contribution to LUAD progression have not, to our knowledge, been previously described [33–36]. Our IP-MS and co-immunoprecipitation data, together with rescue assays, suggest that ZCCHC3 is not merely associated with NCOA5 but functionally required for its full oncogenic activity. Another important contribution of this study is the identification of FGF22 as a direct transcriptional target repressed by NCOA5 in LUAD. Although members of the FGF family have been implicated in lung cancer biology, the role of FGF22 in LUAD remains poorly defined, and its upstream regulation has not been well characterized. Our ChIP-seq, luciferase, and functional rescue data support a model in which NCOA5 promotes LUAD progression, at least in part, by suppressing the tumor suppressive effects of FGF22. This provides a more mechanistically specific explanation for the oncogenic role of NCOA5 in LUAD.

FGFs play complex and context-dependent roles in tumor biology, with different family members exerting either oncogenic or tumor suppressive effects depending on the cellular and microenvironmental context. While many FGFs promote tumor progression through activation of FGF receptor (FGFR) signaling, leading to downstream activation of pathways such as MAPK and PI3K/AKT, emerging evidence suggests that certain FGFs may also exert inhibitory effects under specific conditions [37–40]. In lung cancer, overexpression of FGF proteins and alterations in FGFR genes, such as amplification and mutation, are linked to disease initiation, progression, and treatment resistance, suggesting that targeting the FGF/FGFR pathway could be an effective therapeutic approach. The FGF family members like FGF1, FGF2, and FGF19, which are associated with lung cancer development [40–44]. In glioblastoma and lung cancer, lower expression of FGF22 correlates with better survival, while higher expression in pancreatic ductal adenocarcinoma is linked to better prognosis, indicating FGF22’s varying roles across cancers [37–44]. It has been recognized as a prognostic marker for lung adenocarcinoma, although its specific role in LUAD progression remains unclear [45]. In this study, we identified FGF22 as a potential tumor suppressor in LUAD, as its overexpression inhibited cell proliferation, migration, and invasion, while its knockdown enhanced malignant phenotypes. However, the precise mechanisms underlying this effect remain unclear. It is possible that FGF22 interacts with specific FGFR receptors to modulate downstream signaling cascades, or that it influences tumor progression through paracrine signaling within the tumor microenvironment, potentially affecting stromal or immune cell interactions. Given the known heterogeneity of FGF signaling, further studies will be required to determine the receptor specificity, downstream signaling pathways, and microenvironmental interactions associated with FGF22 in LUAD. Therefore, the role of FGF22 identified in this study should be considered context-dependent and mechanistically exploratory, providing a basis for future investigation.

Importantly, our additional analyses provide further insight into the relationship between NCOA5, ZCCHC3, and FGF22. We observed that ZCCHC3 knockdown increased FGF22 expression and partially reversed the suppressive effect of NCOA5 overexpression on FGF22, suggesting that ZCCHC3 contributes to NCOA5-mediated transcriptional repression. These findings support a model in which ZCCHC3 may act, at least in part, as a functional cofactor of NCOA5, facilitating its regulatory effects on downstream target genes such as FGF22. However, as our conclusions are based primarily on expression and functional assays, we cannot exclude the possibility that ZCCHC3 may also participate in parallel regulatory pathways independent of NCOA5. Further studies, including chromatin immunoprecipitation or transcriptional complex analyses, will be required to fully elucidate the mechanistic role of ZCCHC3 in NCOA5-mediated gene regulation. In vivo experiments with a nude mouse model confirmed that NCOA5 enhanced tumor growth, while co-expressing FGF22 or shZCCHC3 with NCOA5 mitigated this effect, demonstrating that FGF22 can antagonize the oncogenic functions of NCOA5 and ZCCHC3.

Several limitations should be acknowledged. First, this study was conducted using a single-center cohort of 94 LUAD patients, which may introduce selection bias and limit the generalizability of the findings. Although the cohort was well-characterized and supported by long-term follow-up data, validation in larger, multi-center, and ethnically diverse populations will be necessary to confirm the clinical relevance of the NCOA5-ZCCHC3/FGF22 axis. Second, external validation across independent datasets showed variability, underscoring the need for further confirmation in large, multi-center, and standardized cohorts. Our findings are partially supported by analyses of public datasets such as TCGA, which demonstrated consistent expression patterns and strengthened the robustness of our observations, validation using independent GEO cohorts (GSE43458, GSE32863, and GSE118370) yielded partially inconsistent expression patterns. These discrepancies may reflect differences in sequencing platforms, cohort composition, sample size, and technical variability, which are well-recognized challenges in cross-cohort transcriptomic studies of LUAD. Therefore, while our results support a biologically relevant role for the NCOA5-ZCCHC3/FGF22 axis, its prognostic significance should be interpreted with caution, and further validation in large, standardized, multi-center cohorts will be necessary. Third, although we validated key findings in multiple LUAD cell lines, including A549, H1299, and H1975, some mechanistic experiments were primarily performed in A549 cells. Therefore, while our results suggest that the NCOA5-ZCCHC3/FGF22 axis is not cell line–specific, further validation across additional LUAD models will be important to confirm the generalizability of these findings. Finally, while our data support a mechanistic role for the NCOA5-ZCCHC3/FGF22 axis, the precise downstream signaling pathways and molecular interactions remain to be fully elucidated. Taken together, our study provides integrated clinical, molecular, and functional evidence supporting a novel regulatory axis in LUAD, while also highlighting the complexity and context-dependence of its biological and clinical significance.

## Conclusion

In conclusion, our study demonstrates that NCOA5 is clinically relevant and functionally oncogenic in LUAD and further reveals a previously uncharacterized mechanism involving ZCCHC3 interaction and transcriptional repression of FGF22. These findings extend current understanding of NCOA5 beyond its previously reported roles in other malignancies and establish the NCOA5–ZCCHC3/FGF22 axis as a mechanistically relevant pathway in LUAD progression.

## Supplementary information

Supplmantary file.docx

Full and uncropped western blots

## Data Availability

All data and materials generated and analyzed during the present study are available from the corresponding author on reasonable requests.
