# Supplementary material for: Identification and validation of NCOA5-ZCCHC3/FGF22 axis as a prognostic marker in lung adenocarcinoma migration and invasion
Source: Cell Death Dis. 2026 Jul 25;17(1):896. doi: 10.1038/s41419-026-09127-9 (PMC13683410; doi:10.1038/s41419-026-09127-9)

Figure 1B

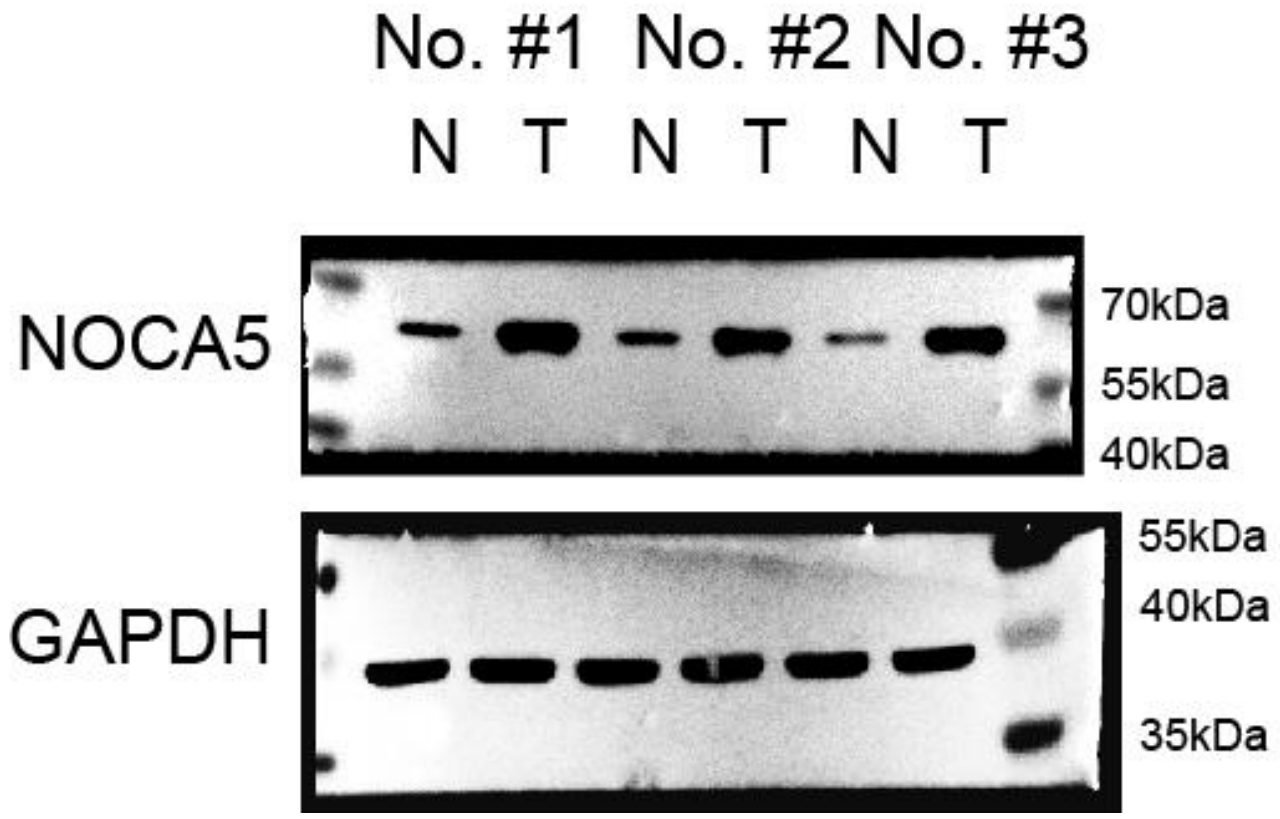

Figure 2A

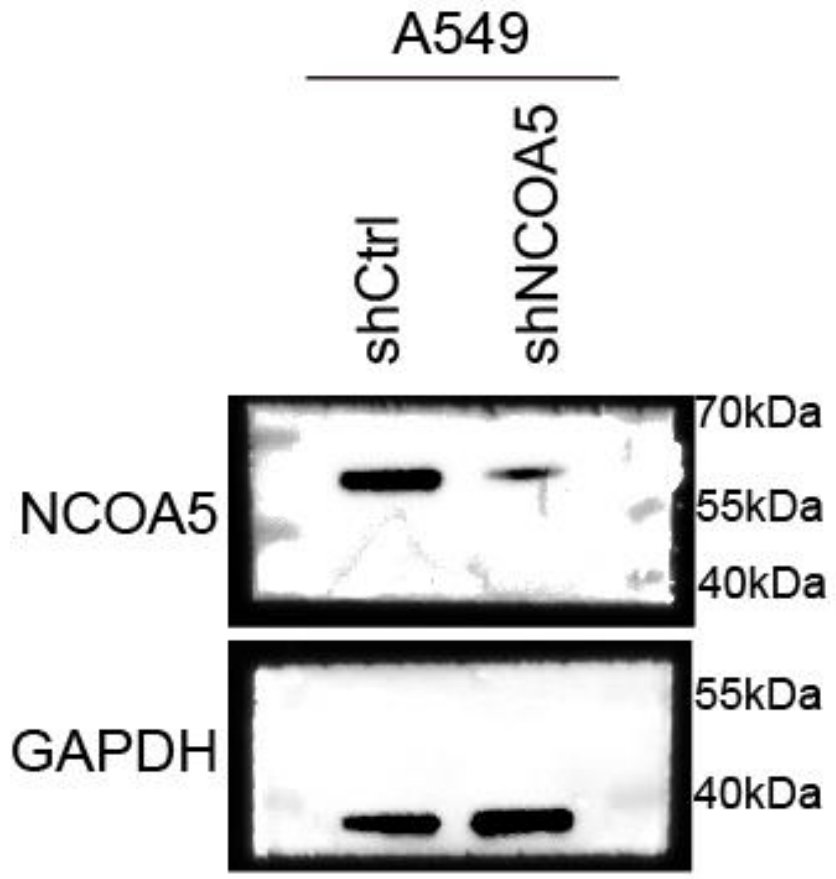

Figure 2F

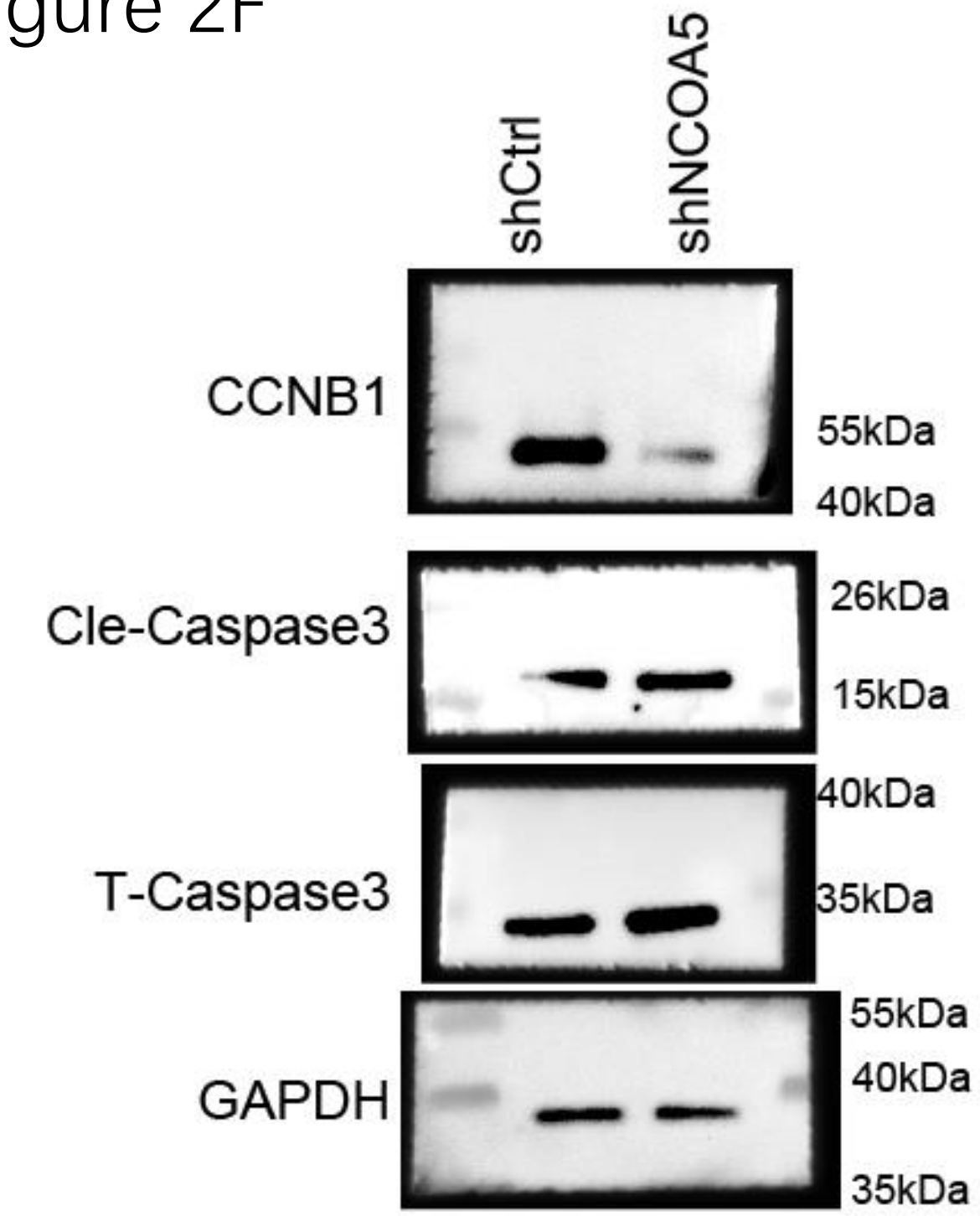

Figure 3A

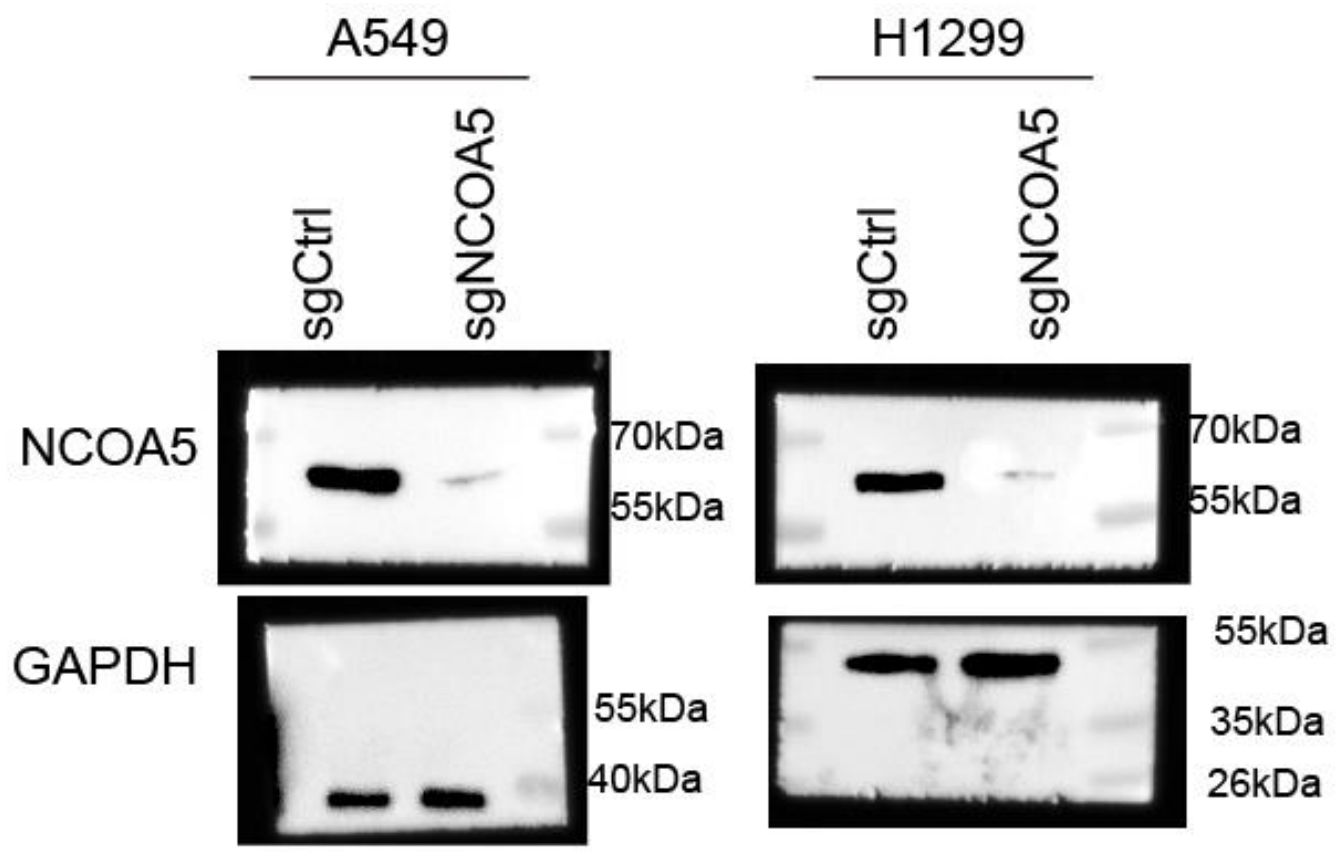

Figure 4A

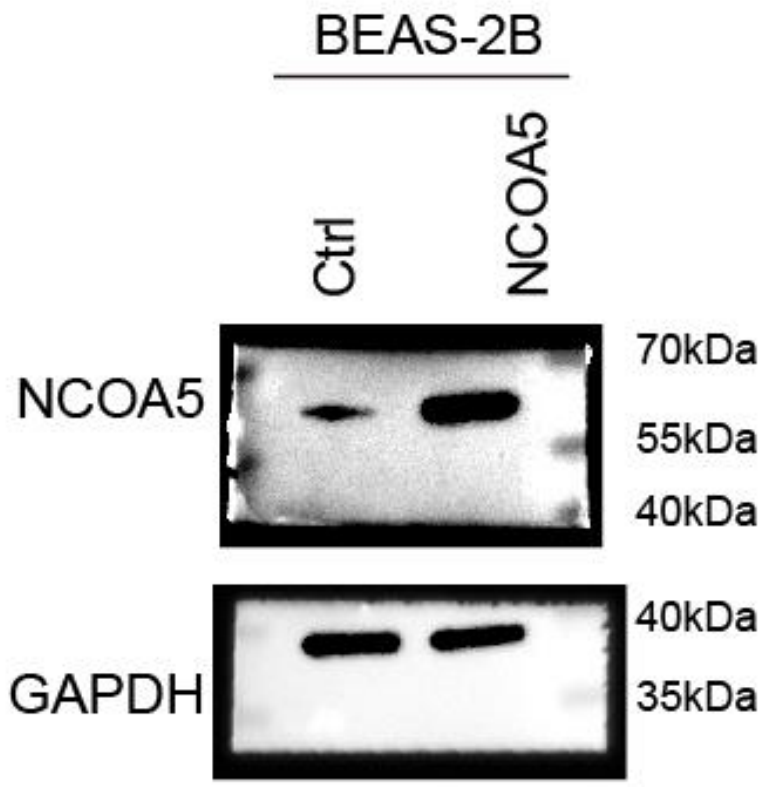

Figure 5B

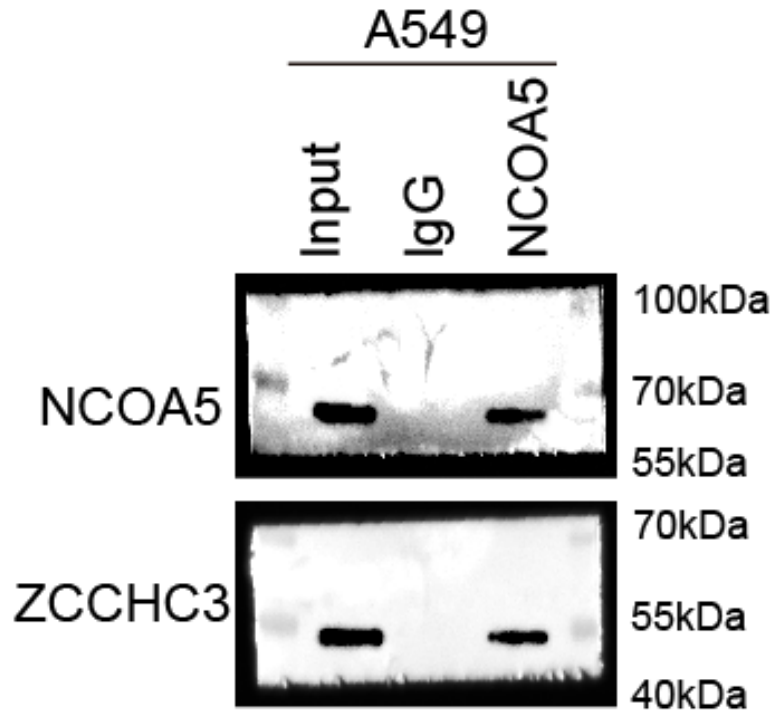

Figure 5C

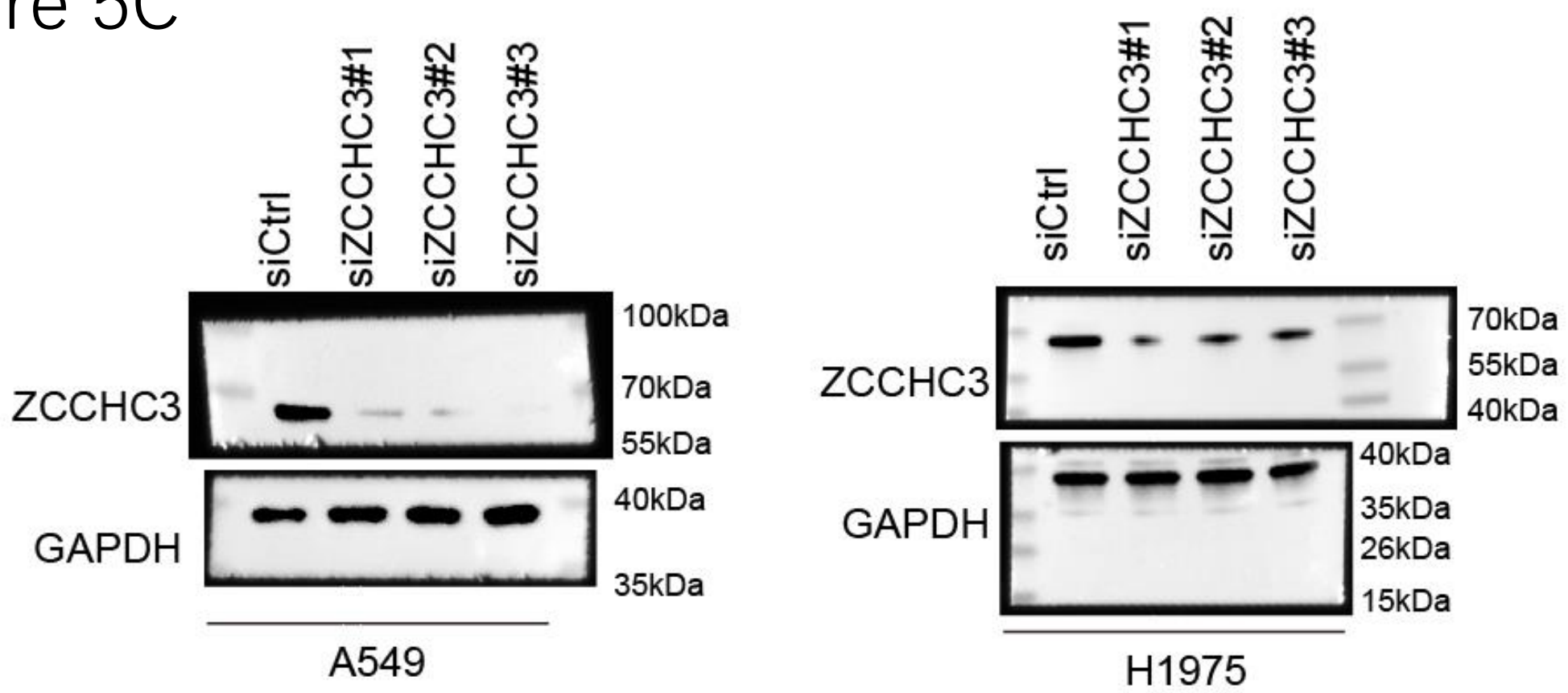

Figure 5G

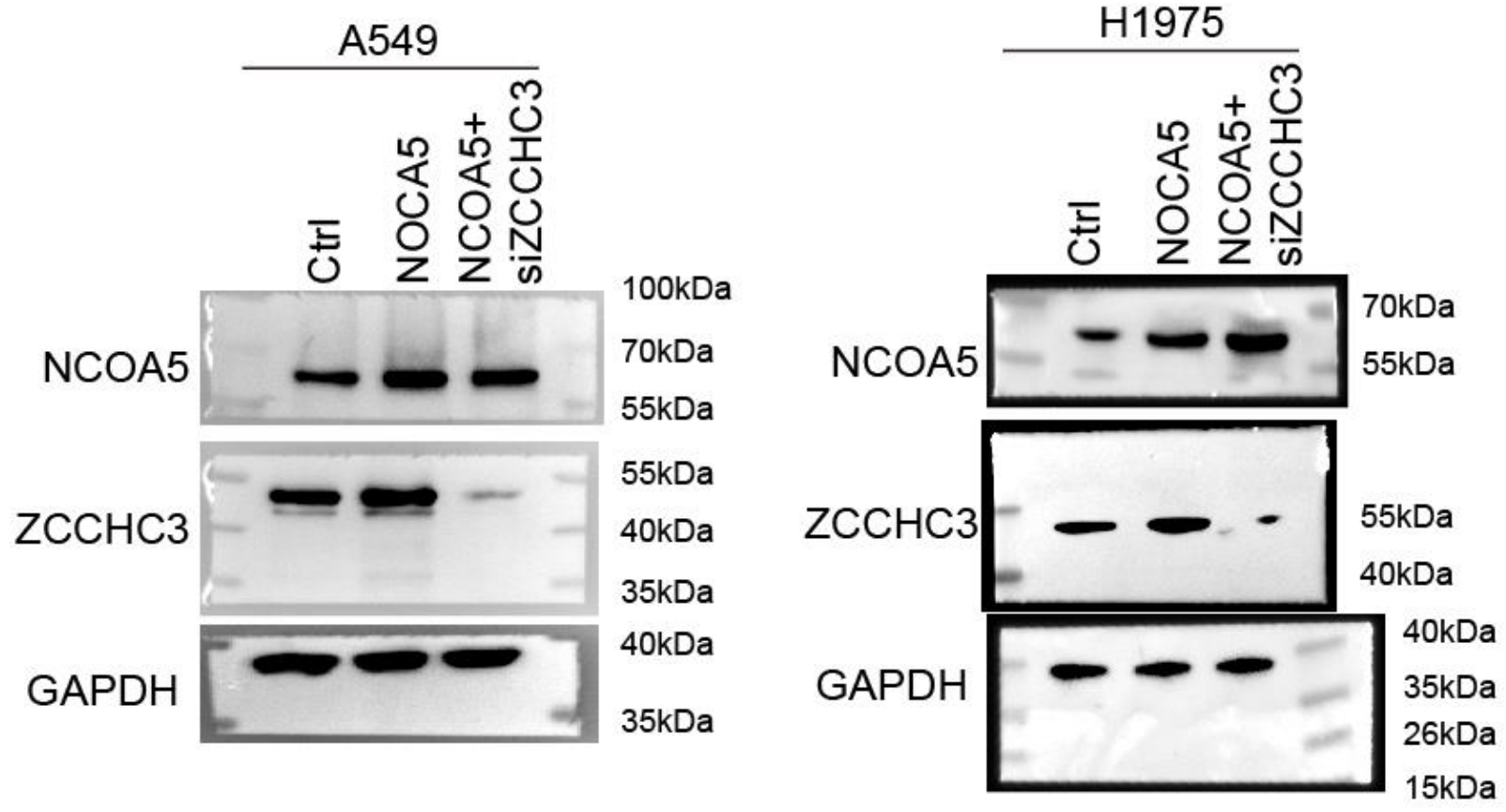

Figure 6I

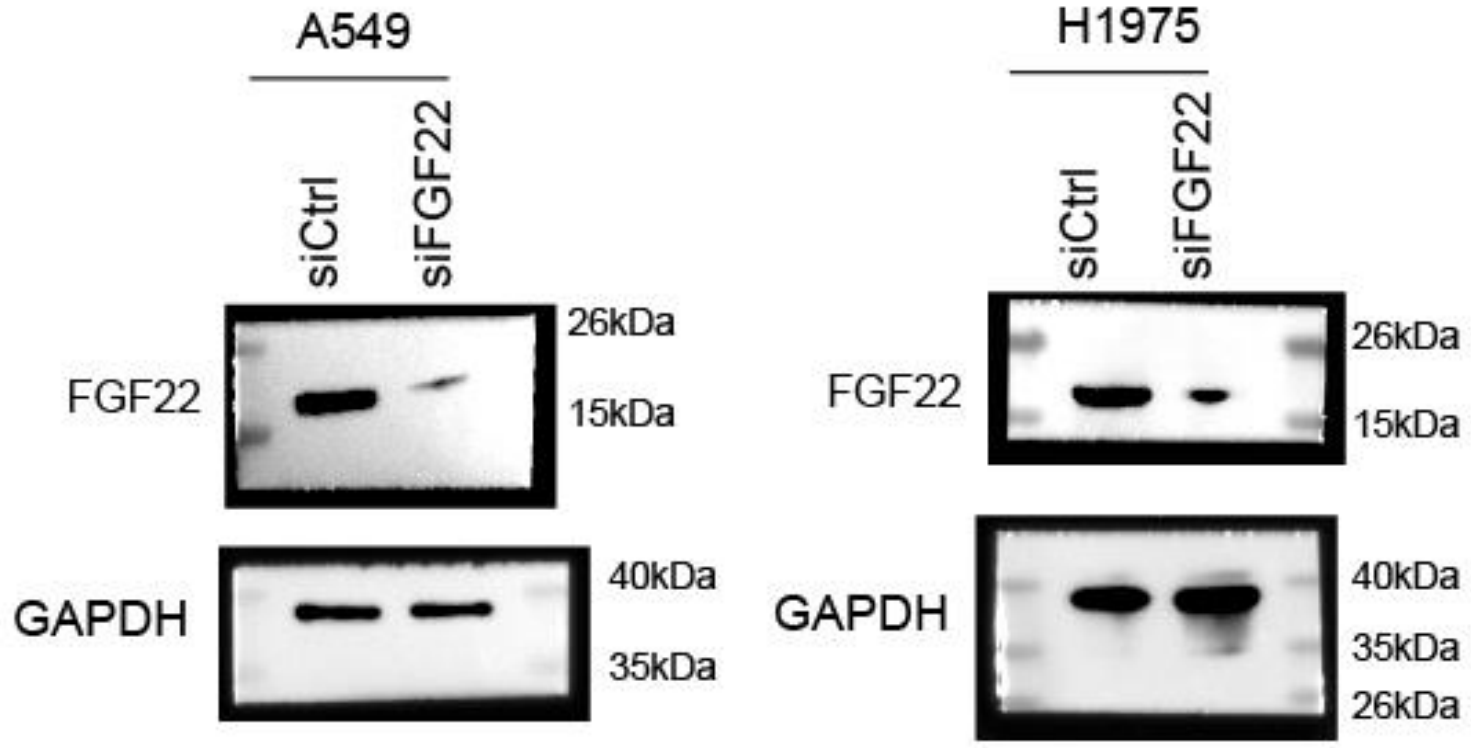

Figure 6N

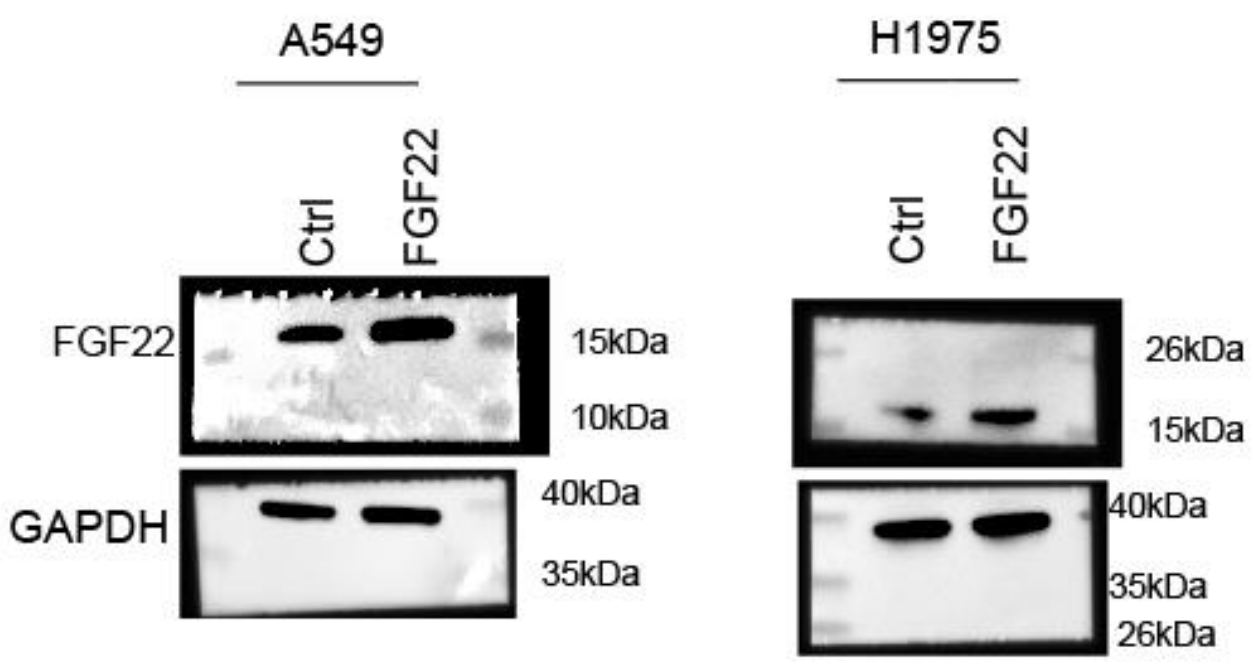

Figure 7A

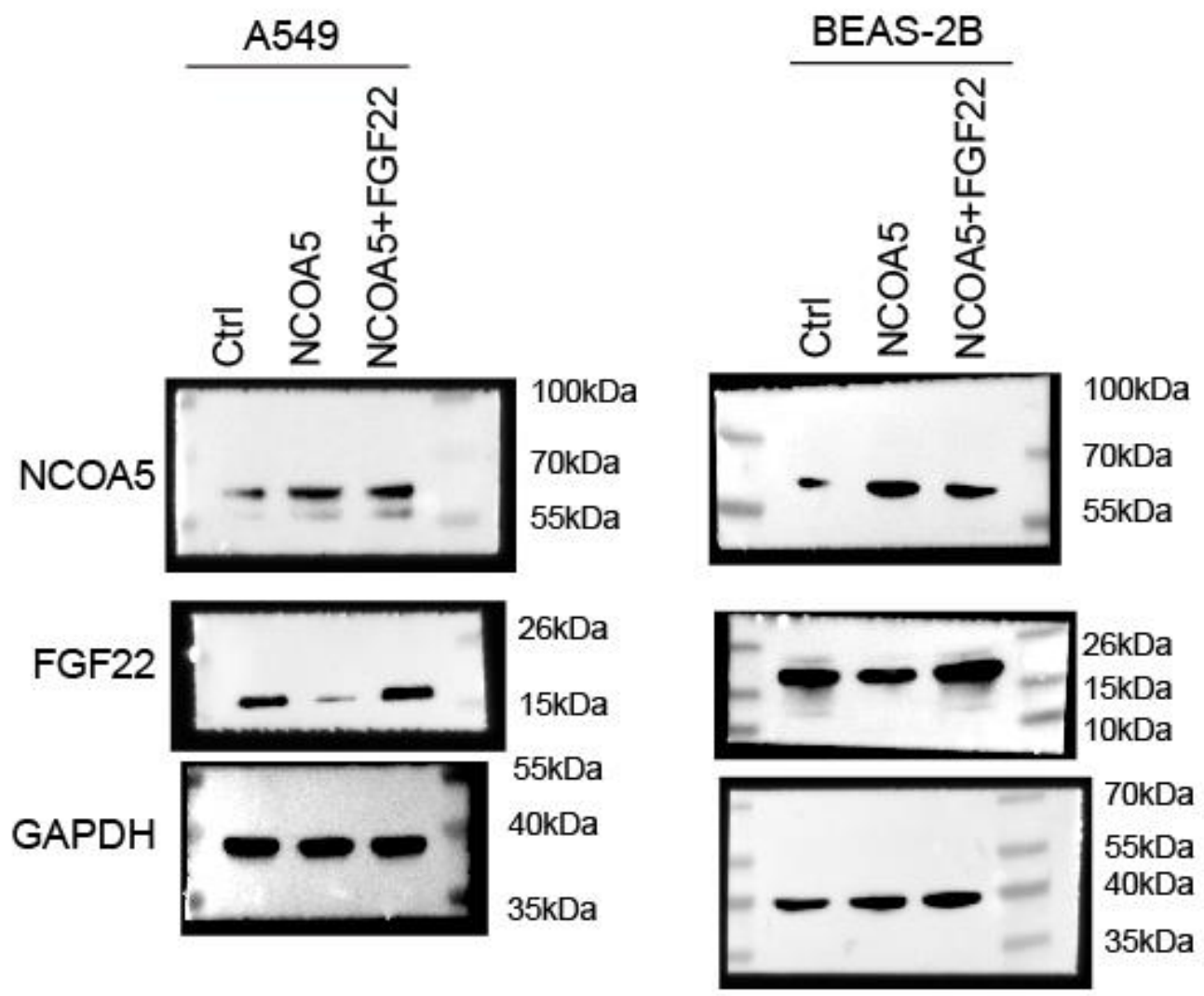

Figure S2B

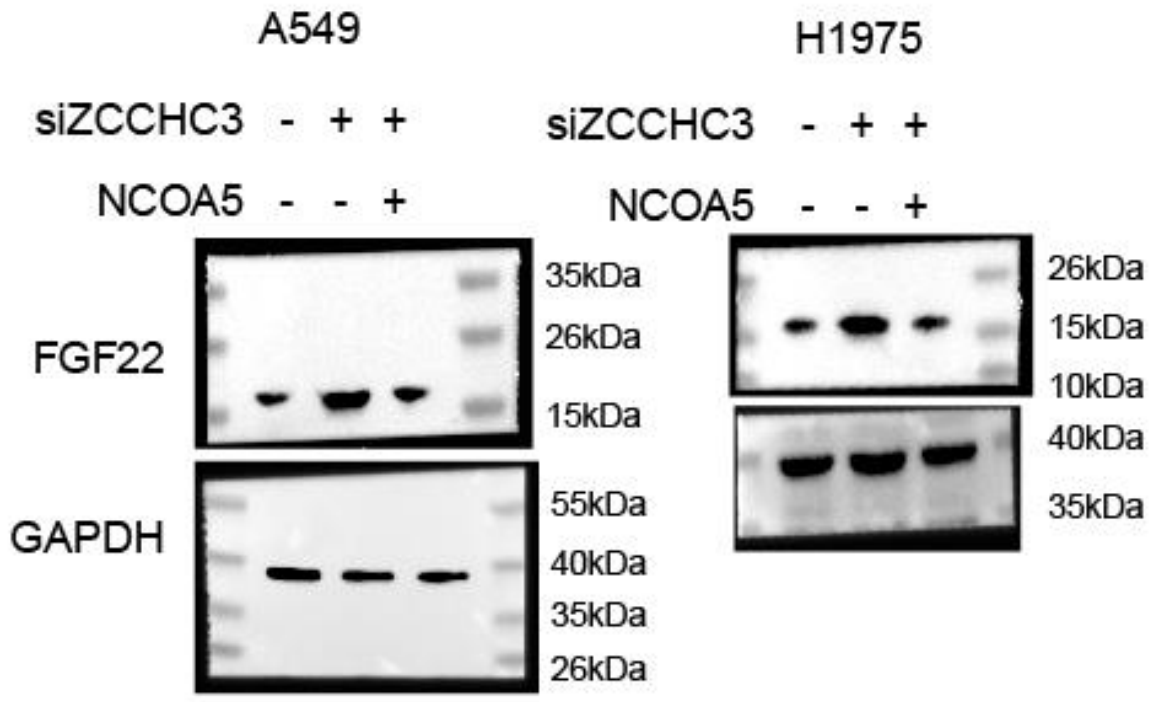

Supplement: Supplementary file 1 [file 41419_2026_9127_MOESM2_ESM.pdf]
