# Supplementary material for: Identification and validation of NCOA5-ZCCHC3/FGF22 axis as a prognostic marker in lung adenocarcinoma migration and invasion
Source: Cell Death Dis. 2026 Jul 25;17(1):896. doi: 10.1038/s41419-026-09127-9 (PMC13683410; doi:10.1038/s41419-026-09127-9)
Supplement: Supplementary file 2 [file 41419_2026_9127_MOESM1_ESM.docx]

Fig S1. TAF15 and ZNF326 did not detect an interaction with NCOA5. Co-immunoprecipitation assays validated TAF15 and ZNF326 do not interacted with NCOA5.

Fig S2. ZCCHC3 participates in NCOA5-mediated repression of FGF22. Knockdown of ZCCHC3 significantly increased FGF22 mRNA and protein levels, NCOA5 overexpression markedly reduced FGF22 expression as demonstrated by RT-qPCR and Western blotting(^***^P<0.001).

Fig S3. Expression Validation of NCOA5, FGF22 and ZCCHC3 in GEO Datasets.

Table S1 Antibodies

| NCOA5 | 1:1000, Protein Tech, 20175-1-AP |
| --- | --- |
| ZCCHC3 | 1:1000, Protein Tech, 29399-1-AP |
| FGF22 | 1:1000, Abcam ab74860 |
| GAPDH | 1:2,500, Abcam ab9485 |
| CCNB1 | 1:1000, Protein Tech, 55004-1-AP |
| Caspase 3 | 1:1000, Protein Tech, 25128-1-AP |
| TAF15 | 1:1000, Protein Tech, 82860-1-RR |
| ZNF326 | 1:1000, Protein Tech, 25147-1-AP |
| Flag | 1:1000, Protein Tech, 66008-4-Ig |
| GAPDH | 1:2,500, Abcam ab9485 |

Table S2 Reagents

| RIPA lysis buffer | Beyotime |
| --- | --- |
| PVDF membrane | Millipore |
| Trizol Reagent | Invitrogen |
| PrimeScript RT kit | Takara |
| SYBR Premix Ex Taq kit | Takara |
| ABI 7500 system | Applied Biosystems |
| DMEM | Gibco-Invitrogen |
| FBS | Hyclone Company |
| EDTA | Gibco-Invitrogen |
| Lipofectamine 2000 | Invitrogen |
| RNase A | Sigma |
| PI | Sigma |
| Annexin V-APC | absin, Shanghai |
| 7-AAD | absin |
| Transwell assays | Corning, NY, USA |
| Matrigel | Mogengel Bio #082704, Xiamen, China |
| Lung Cancer Organoid Kit | Cat# L0519-01, VentureBioGen |
| Primary Tissue Storage Solution | K601005, bioGenous |
| Tumor Tissue Digestion Solution | K601003, bioGenous |
| Cancer Organoid Basal Medium | B213152, bioGenous |
| Organoid Culture ECM | M315066, bioGenous |
| NE-PER™ Nuclear and Cytoplasmic Extraction Reagents | Thermo Fisher Scientific |
| A/G agarose beads | Thermo Fisher Scientific |
| NETN buffer | ED-9024, Ecotop Scientific |
| EASY nLC 1200-Orbitrap Fusion Lumos + ETD system | Thermo Fisher Scientific |
| A/G agarose beads | Santa Cruz Biotechnology, Inc. |
| pGL4 Luciferase Reporter Vector | Promega |
| Lipo8000 | Beyotime |
| Luciferase Reporter Gene Assay Kit | Beyotime |
| microplate spectrophotometer | Bio-Rad |
|  |  |

Table S3 Primers

| NCOA5: | forward 5’-ATGAATACGGCTCCATCAAGACC-3’, |
| --- | --- |
|  | reverse 5’-ACTTCCTCGAATTGGGGATCG-3’, |
| ZCCHC3: | forward 5’-CAAGTCCAGCTTGAAGACGCT-3’, |
|  | reverse 5’-CCAAACCTGTCGGTCACTTTC-3’, |
| FGF22: | forward 5’-GGGAGCGCATCGAAGAGAAC-3’, |
|  | reverse 5’-CTGTGAGGCGTAGGTGTTGTG-3’, |
| GAPDH (endogenous control): | forward 5’-GTGGGCATCAATGGATTTGG-3’, |
|  | reverse 5’-ACACCATGTATTCCGGGTCAAT-3’, |
